# Computational Insight into Intraspecies Distinctions in *Pseudoalteromonas distincta*: Carotenoid-like Synthesis Traits and Genomic Heterogeneity

**DOI:** 10.3390/ijms24044158

**Published:** 2023-02-19

**Authors:** Larissa Balabanova, Olga Nedashkovskaya, Nadezhda Otstavnykh, Marina Isaeva, Oksana Kolpakova, Iuliia Pentehina, Aleksandra Seitkalieva, Yulia Noskova, Varvara Stepochkina, Oksana Son, Liudmila Tekutyeva

**Affiliations:** 1 Advanced Engineering School, Institute of Biotechnology, Bioengineering and Food Systems, Far Eastern Federal University, 10 Ajax Bay, Russky Island, 690922 Vladivostok, Russia; 2 Laboratory of Marine Biochemistry, G.B. Elyakov Pacific Institute of Bioorganic Chemistry, Far Eastern Branch, Russian Academy of Sciences, Prospect 100-Letya Vladivostoka 152, 690022 Vladivostok, Russia; 3 ARNIKA, Territory of PDA Nadezhdinskaya, Centralnaya St. 42, Volno-Nadezhdinskoye, Primorsky Krai, 692481 Vladivostok, Russia

**Keywords:** marine bacteria, *Pseudoalteromonas*, phenotype variability, phylogenomic relationships, pan-genome, biosynthetic gene clusters, aryl polyene hybrid, metabolic pathways, metabolic reconstruction

## Abstract

Advances in the computational annotation of genomes and the predictive potential of current metabolic models, based on more than thousands of experimental phenotypes, allow them to be applied to identify the diversity of metabolic pathways at the level of ecophysiology differentiation within taxa and to predict phenotypes, secondary metabolites, host-associated interactions, survivability, and biochemical productivity under proposed environmental conditions. The significantly distinctive phenotypes of members of the marine bacterial species *Pseudoalteromonas distincta* and an inability to use common molecular markers make their identification within the genus *Pseudoalteromonas* and prediction of their biotechnology potential impossible without genome-scale analysis and metabolic reconstruction. A new strain, KMM 6257, of a carotenoid-like phenotype, isolated from a deep-habituating starfish, emended the description of *P. distincta*, particularly in the temperature growth range from 4 to 37 °C. The taxonomic status of all available closely related species was elucidated by phylogenomics. *P. distincta* possesses putative methylerythritol phosphate pathway II and 4,4′-diapolycopenedioate biosynthesis, related to C30 carotenoids, and their functional analogues, aryl polyene biosynthetic gene clusters (BGC). However, the yellow-orange pigmentation phenotypes in some strains coincide with the presence of a hybrid BGC encoding for aryl polyene esterified with resorcinol. The alginate degradation and glycosylated immunosuppressant production, similar to brasilicardin, streptorubin, and nucleocidines, are the common predicted features. Starch, agar, carrageenan, xylose, lignin-derived compound degradation, polysaccharide, folate, and cobalamin biosynthesis are all strain-specific.

## 1. Introduction

The description of the marine bacterial species *Pseudoalteromonas distincta* is constantly being revised due to the high intraspecific phenotypic and genomic differentiation among the isolates collected from different marine habitats [1]. Until recently, they were classified as the different species belonging to the highly polyphyletic genus *Pseudoalteromonas*, such as *Pseudoalteromonas haloplanktis* (the strains AC163, TB25, TAE79, TAE80, ANT/505), and *Pseudoalteromonas elyakovii* (the strain SM1926) (NCBI genome IDs: GCA_000497935.1, GCA_000497995.1, GCA_000498015.1, GCA_000498035.1, and GCA_000212655.3). The genome-based analysis implemented by the EzBioCloud tools allowed them to be clustered into the single species *P. distincta* [2]. The species *Pseudoalteromonas paragorgicola* with the single strain KMM 3548^T^ has also been recently reclassified as *P. distincta* KMM 3548 [1]. The *P. distincta* strains were isolated from predominantly cold marine environments, including sponges, gorgonian, water columns, sediments, and brown algae surfaces [3,4,5]. The representatives of the species *P. distincta* possess strictly aerobic, Gram-stain-negative, non-spore-forming cells that may motile by means of single polar or four to seven lateral flagella [1]. The agar-plated colonies of the *P. distincta* isolates are either non-pigmented or carotenoid-type colored, from yellow to slight orange, as in the case of the strain *P. distincta* KMM 3548 (formerly *P. parogorgicola* KMM 3548^T^) associated with a gorgonian *Paragorgia arborea* from the Pacific Ocean. However, the type strain *P. distincta* KMM 638^T^ (=ATCC 700518^T^), isolated from a marine sponge collected at a depth of 350 m near the Komandorskie Islands, produces diffusible melanin-like pigments [1]. According to EzBioCloud, there are currently ten whole-genome sequenced *P. distincta* strains, but not all of them have been validated as belonging to the species [2].

The bacteria of the genus *Pseudoalteromonas* themselves are highly polyphyletic due to their ability to adapt to the local marine econiches, which are characterized by harsh chemical/physical conditions such as, for example, very low/high temperatures, and produce chemically different secondary metabolites of biotechnological relevance, including algal polysaccharide-degrading enzymes and biologically active compounds with strong antimicrobial and antitumor activities [1,4,5,6,7]. The *Pseudoalteromonas* pangenome, computed by Bosi et al. [4], included two main *Pseudoalteromonas* groups: the pigmented and non-pigmented strains. The authors highlighted a remarkable genomic heterogeneity even for the closely related *Pseudoalteromonas* isolates [4]. It has been suggested that pigmentation in *Pseudoalteromonas* spp., particularly isolated from marine invertebrates, is associated with genome enrichment in the hybrid gene clusters encoding for nonribosomal peptide synthetases and polyketide synthases (NRPS/PKS) and the production of bioactive compounds, such as alterochromide, pseudoalterobactin, alteramide A, thiomarinols, indolmycin, pentabromopseudilin, and pseudochelin [7].

In general, marine pigmented bacteria are known to be a promising source of melanins and carotenoids as natural high antioxidant efficiency compounds with rare structures, as well as a plethora of other highly specific secondary metabolites with biotechnology and therapy applications [8,9,10,11,12]. Carotenoids are isoprenoid derivatives, characterized by a polyene chain (C30, C40, or C50), with conjugated double bonds providing distinctive absorption patterns and coloration. They protect microbial pathogens from UV radiation and host immune system-generated oxygen radicals [13,14,15,16,17]. The symbiotrophic gut microbiota is reported to contribute significantly to retinoid biosynthesis (vitamin A), which is important for the host’s health [15,18,19]. Currently, there are more than 700 types of carotenoids, and their biosynthesis pathway signatures are partially or completely established, allowing genomic data mining to reveal and characterize them [10,13,15,20]. However, the host-associated γ-*Proteobacteria* and *Bacteroidetes* (both pathogens and gut microbiota) have been found to possess additional or alternative gene clusters for biosynthesis of membrane-bound yellow pigments, aryl polyenes of polyisoprenoid structures with polyene moiety, which are functionally analogous to the antioxidative carotenoids and contribute to biofilm formation, increased virulence, epiphytic survival, host-pathogen interactions, and symbiosis [21,22,23,24,25]. Melanins are also distributed as oxygen radical scavengers and virulence factors among microorganisms and are formed by the oxidative polymerization of phenolic or indolic compounds [8,26]. A lot of marine bacterial pigments were investigated for antimicrobial, anti-inflammatory, antioxidant, anti-HSV-1, anticancer, antidiabetic, and wound healing activities, indicating their potential biomedical applications as well as food, feed, cosmetic, and pharmaceutical formulations [8,9,12]. In addition, the natural marine pigments and other secondary metabolites could be used as “green chemistry” to replace synthetic compounds [11,12,26,27,28,29,30].

Herein, we analyzed the publicly available and novel sequenced genomes of the strains putatively belonging to the species *P. distincta* isolated from diverse ecological niches, such as marine water, invertebrates, and algae. In order to elucidate their species-specific features and taxonomic state among all other *Pseudoalteromonas* species, the type strain of the species *Pseudoalteromonas elyakovii* had to be sequenced and deposited in GenBank due to the absence of its valid genome in the database. The non-distinguished phenotypes, chemotaxonomy, protein sequences, and metabolic pathways for *P. distincta* and *Pseudoalteromonas arctica* required the genome-based species delineation. The whole-genome sequences were used to decipher *P. distincta*’s contribution to the *Pseudoalteromonas* pangenome; to reconstruct its strain-specific metabolic pathways for central and secondary metabolism, particularly carotenoid- and melanin-like pigments and polysaccharides; and to predict the orthologous Biosynthetic Gene Clusters encoding for bioactive compounds (BGCs), as well as Genomic Islands (GIs), determining their remarkable intraspecies genomic and phenotypic heterogeneity due to the local environmental adaptation and rapid diversification of species.

## 2. Results and Discussion

### 2.1. Phenotypic Characterization of the P. distincta Strains

**Figure 1:**
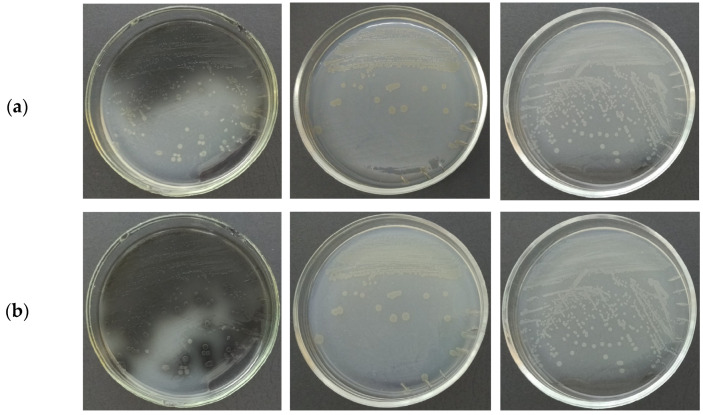
The strains *P. distincta* KMM 638^T^ (left, diffusible black pigmentation), *P. distincta* KMM 3548 (formerly *P. paragorgicola* KMM 3548) (middle, cell-bound slight yellow-orange pigmentation), and *P. distincta* KMM 701 (right, whitish) were cultivated on marine agar under the daylight for 7 (**a**) and 9 (**b**) days at 24 °C.

Along with the presence of similarity in physiological and biochemical features, significant phenotypic variability was observed among the strains of *P. distincta* studied here (Table 1). They formed whitish (strains KMM 638^T^ and KMM 701) and light-orange (KMM 3548 and KMM 6257) colonies on marine agar. Moreover, the strain *P. distincta* KMM 638^T^, isolated from a sponge, produced a diffusible melanin-like pigment on the fifth day of cultivation (Figure 1). The synthesis levels of carotenoid- and melanin-like pigments in *P. distincta* were significantly dependent on the light during cultivation. Based on the distinct phenotypes and 16S rRNA gene sequences, the melanin-producing strain KMM 638^T^ and the strain KMM 3548, with the carotenoid-like synthesis traits, were described as different species in the past, but later they combined into one single species, *P. distincta* [1,3,31]. The study of phenotypic characteristics of the recently discovered strain KMM 6257 (=2-2A-13) isolated from a starfish as a novel member of the species *P. distincta*, which produces slightly yellow-orange pigments, extended the temperature ranges for growth for this species to 4–37 °C (Table 1). Other strain-dependent phenotypic characteristics are shown in Table 1. However, the strains of *P. distincta* share similar phenotypic traits with their closest relative type strain, *Pseudoalteromonas arctica* A 37-1-2^T^, including carotenoid-like pigmentation (Table 1) and the presence of a 16S rRNA sequence with 100% identity with the genes of *P. distincta* (Table S1). Thus, the issue of *P. arctica* A 37-1-2^T^ belonging to the species *P. distincta* required different methods to be applied in order to delineate or reclassify these two species.

The *P. distincta* and *P. arctica* A 37-1-2^T^ strains had many phenotypic properties in common. Thus, all of them were strictly aerobic and motile bacteria that degraded aesculin and produced acid from sucrose; they also could not reduce nitrate to nitrite, hydrolyze agar and urea, oxidize L-arabinose, fructose, mannose, L-rhamnose, ribose, trehalose, *N*-acetylglucosamine, and glycerol, and assimilate L-arabinose, L-fucose, L-rhamnose, and *N*-acetylglucosamine. The majority of strains were characterized by the presence of oxidase, catalase, alkaline phosphatase, esterase lipase (C8), leucine arylamidase, valine arylamidase, leucine arylamidase, acid phosphatase, and naphthol-AS-BI-phosphohydrolase activities; and the absence of chitin degradation and CM-cellulose, and the assimilation of subaric acid, potassium 5-ketogluconate, 3-hydroxybenzoique acid, 4-hydroxybenzoique acid, and salicin; taken together with lipase (C14), *α*-galactosidase, *β*-galactosidase, *β*-glucuronidase, *α*-glucosidase, *β*-glucosidase, *N*-acetylglucosaminidase, *α*-mannosidase, and *α*-fucosidase activities. The prevalent strains of *P. distincta* could hydrolyze casein and gelatin, produce acid from cellobiose and maltose, and utilize citrate. A part of the above tests was not indicated for *P. arctica* A 37-1-2^T^, because it was not studied using API 32 GN and API ZYM strips and susceptibility to antibiotics [32].

The fatty acid composition of three strains of the species *P. distincta* was also similar to *P. arctica* A 37-1-2^T^ (Table 2). The presence of prevalent fatty acids in the novel strain KMM 6257 (=2-2A-13) and *P. arctica* A 37-1-2^T^, such as C_16:1 ω*7c*_ (29–40.2%), C_16:0_ (12.7–18.2%), C_17:1 ω*8c*_ (15.3–17.9%), C_18:1 ω*7c*_ (5.2–11%), and C_12:0_ 3-OH (4.8–7.5%), is in line with the data reported previously for *P. distincta* [1] except that *P. arctica* A 37-1-2^T^ contained a lower proportion of C_17:0 and_ C_17:1 ω*8c*_ (Table 2).

### 2.2. Phylogenomic Relationships of the P. distincta Strains within the Genus Pseudoalteromonas

The delineation of the species within the genus *Pseudoalteromonas* remains difficult because of their diverse breakdown pattern of carbohydrates, the lack of useful chemotaxonomic markers, and the increased copy numbers of their 16S rRNA genes (Table S1), which have an insufficient degree of sequence similarity even within a strain [1,33]. To date, a polyphasic approach has been widely applied for the identification of environmental isolates, which along with chemotaxonomic and phenotypic methods includes phylogenomic analysis and evaluation of the genome relatedness indexes, such as Average Nucleotide Identity (ANI), Amino Acid Identity (AAI), and digital DNA-DNA hybridization (dDDH). Thereby, a remarkable genomic heterogeneity both at the genus and group levels has been shown even for the closely related strains on the basis of a comprehensive comparative genomic analysis for a large portion of the *Pseudoalteromonas* strains [4].

**Figure 2:**
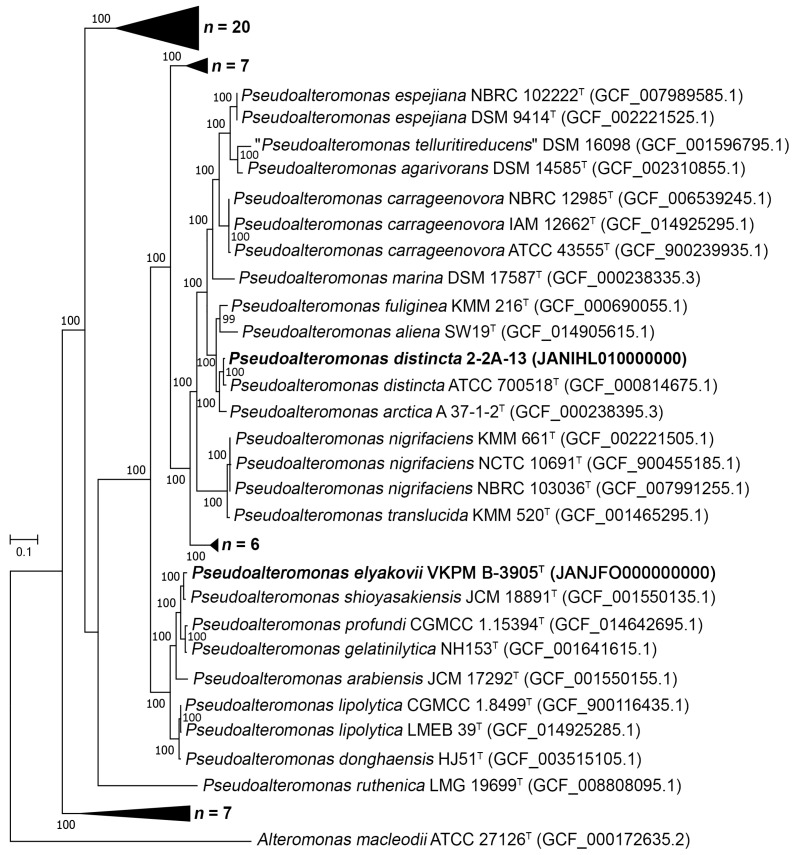
Maximum-likelihood phylogeny of the genus *Pseudoalteromonas* based on 400 universal markers selected by PhyloPhlAn3.0 and reconstructed by RAxML with non-parametric bootstrapping using 100 replicates, including Bar, with 0.1 substitutions per amino acid position. The corresponding GenBank accession numbers for genomes are given in parentheses. Representatives with novel genomes are in bold.

To clarify the phylogenomic relationships of the new strain 2-2A-13, a phylogenetic tree of its genome together with 65 genomes of the type strains from the genus *Pseudoalteromonas* was built using PhyloPhlAn3.0 based on 400 concatenated proteins [34]. The strain 2-2A-13 clustered together with the type strain *P. distincta* ATCC 700518^T^ (Figure 2; Supplementary Figure S1, Table S2).

The ANI, AAI, and dDDH values between the strains 2-2A-13 and ATCC 700518^T^ were 98.12%, 97.99%, and 82.4%, respectively. The obtained ANI and dDDH relatedness values were above thresholds of 95% and 70%, respectively, which are recommended for species delineation [35]. These data clearly show that the strain 2-2A-13 represents the species *P. distincta* (Figure 2; Supplementary Figure S1, Table S2).

Due to the absence of valid genome for the species *Pseudoalteromonas elyakovii* in GenBank, the strain *P. elyakovii* VKPM B-3905^T^ was sequenced and deposited as the species’ type strain under the accession number JANJFO010000000. According to the phylogenomic tree, the type strain *P. elyakovii* VKPM B-3905^T^ formed a well-supported (bpp = 100) separate branch within the clade with the type strain *P. shioyasakiensis* JCM 18891^T^ (Figure 2; Supplementary Figure S1, Table S2). The ANI, AAI, and dDDH values between *P. elyakovii* VKPM B-3905^T^ and *P. shioyasakiensis* JCM 18891^T^ were 96.78%, 97.22%, and 73.6%, respectively. The obtained ANI and dDDH relatedness values were above thresholds of 95% and 70%, respectively, which is recommended for species delineation [35,36]. Thus, the species *P. elyakovii* and *P. shioyasakiensis* rather belong to a single species (Figure 2; Supplementary Figure S1, Table S2). In addition, the species *P. elyakovii* was described in 2000 and *P. shioyasakiensis* in 2014 [33,37]. Otherwise, the *Pseudoalteromonas* branches in the reconstructed genome-wide phylogenetic tree (Supplementary Figure S1, Table S2) coincide in their positions relative to each other with those obtained by Bosi et al. [4].

**Figure 3:**
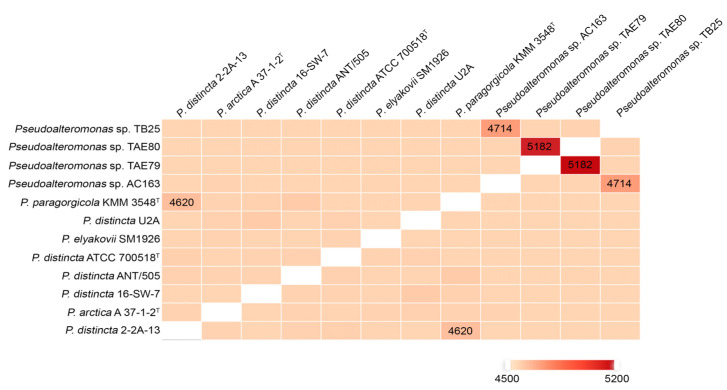
Similarity matrix for pairwise genome comparisons plotted by OrthoVenn2: the heatmap shows the ortholog clusters between any pair of genomes.

As for the species *P. distincta*, the Comparative Genomics (CG) and genomic statistics calculated by EzBiocloud have identified the additional *P. distincta* genomes among the publicly available ones, deposited in the GenBank (NCBI) under the different species titles (Figure 3; Supplementary Figure S2, Table S3).

EzBioCloud is known to be an analytics portal focusing on taxonomy, ecology, genomics, metagenomics, and the microbiome of bacteria and archaea [2]. However, the genomes of *P. distincta* ATCC 700518^T^ and *P. flavipulchra* ATCC BAA-314^T^ clearly represent the same strain since they share 99.99% of ANI, 99.99% of AAI, and 99.9% of dDDH. Thus, the genome of *P. flavipulchra* ATCC BAA-314^T^ was excluded from the analysis (Supplementary Table S3). The genome of *P. paragorgicola* KMM 3548^T^ (GCF_014918315.1) was assigned to *P. distincta* according to its recent reclassification by Nedashkovskaya et al. [1] and added to the analysis from GenBank (NCBI) (Supplementary Table S2). In total, eleven strains have been validly identified to belong to the species *P. distincta*, with their most closely related species *P. arctica* (Figure 2 and Figure 3; Supplementary Figure S2).

### 2.3. Pan-Genomic Characterization of the P. distincta Strains

**Figure 4:**
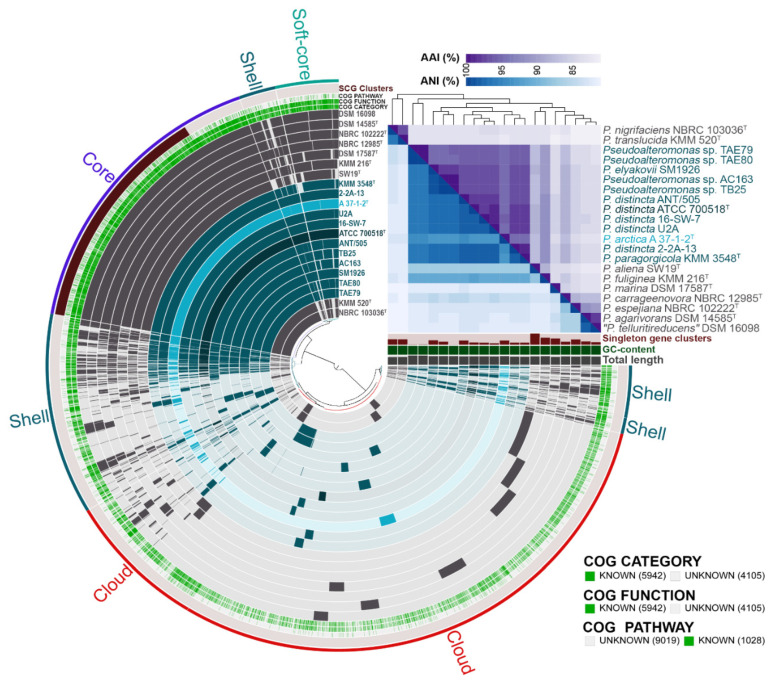
The pan-genome of 21 *Pseudoalteromonas* strains. Clustering of the genomes based on the presence/absence patterns of 10,045 pan-genomic clusters. The genomes are organized in radial layers as core, soft-core, shell, and cloud gene clusters (Euclidean distance; Ward linkage), which are defined by the gene tree in the center. The genome of the type strain *P. distincta* ATCC 700518^T^ is colored very dark blue (hex code #04353d), the genomes of other *P. distincta* strains are colored sherpa blue (hex code #065563), the genome of *P. arctica* A 37-1-1^T^ is colored cyan blue (hex code #10abc7), and the genomes of other *Pseudoalteromonas* species are colored grey (hex code #4f4a4f). The heatmap displays pairwise values of average nucleotide (ANI) and amino acid (AAI) identities in percentages calculated using the online server ANI/AAI-Matrix.

In addition, a pan-genome analysis was performed to investigate more precisely the relationships between the strains of *P. distincta* and the closely related *Pseudoalteromonas* species (Figure 4). The pangenome contained eleven genomes of *P. distincta*, including the novel strain 2-2A-13, and ten genomes of the type strains of the closely related species from the same clade on the phylogenomic tree (Figure 2 and Figure 4).

Overall, the constructed partial *Pseudoalteromonas* pan-genome was divided into core, soft-core, shell, and cloud genes, as follows: core genes were presented in all 21 genomes; soft-core genes were found in 95% of the genomes; shell genes were found in more than two genomes and less than 95% of the genomes; cloud genes were found in no more than two genomes. Of 10,045 gene clusters with 84,968 genes, the *Pseudoalteromonas* pan-genome core comprises 2219 gene clusters, with a soft-core genome containing 469 gene clusters, the accessory genome containing 2316 gene clusters in the shell, and 5041 in the cloud (Figure 4).

The obtained intraspecies ANI, AAI, and dDDH values among the *P. distincta* strains were found to be 95.67−100%, 94.57−100% and 63.60−100%, respectively. These values are above the thresholds of 95% ANI and 70% dDDH, which are recommended for species delineation, with the exception of the lower dDDH value for *P. distincta* KMM 3548 [35,36]. Such significantly diverse dDDH values for the *P. distincta* strains could be explained by the different levels of their genome assemblies [1]. The ANI, AAI, and dDDH values between one *P. arctica* A 37-1-1^T^ and eleven *P. distincta* strains were 93.29–94.25%, 94.70–95.51%, and 52.7–56.9%, respectively. Whereas those values between *P. arctica* A 37-1-1^T^ and representatives of other *Pseudoalteromonas* species were 81.98–90.64%, 83.49–91.66%, and 25–39.1%, respectively. It is worth noting that among the genomes, only the *P. distincta* 16-SW-7 (GCF_005877035.1) and *P. arctica* A 37-1-1^T^ (GCF_000238405.3) genomes have been finished into two circular chromosomes and one plasmid in *P. arctica* A 37-1-1^T^. Nevertheless, the obtained ANI and AAI values for *P. arctica* A 37-1-1^T^, presented on the heatmap in Supplementary Table S4, are below the thresholds of the recommended 95% ANI and 70% dDDH [35,36].

According to the recommendation for species delineation, *P. arctica* and *P. distincta* indeed present separate species, but with the traits of recent diversification and incomplete divergence from an ancestral clonal population (Figure 3 and Figure 4). It is evident from their wide-genome phylogenetic close relatedness and high genomic heterogeneity of *P. distincta* (Figure 3 and Figure 4, Supplementary Figure S2). Indeed, the strains *P. distincta* ATCC 700518^T^ and *P. distincta* SM1926 (=*P. elyakovii* SM1926), as well as *P. arctica* A 37-1-1^T^, equally have lower-similarity patterns with others at their pairwise genome comparisons due to the absence of isolates, which are similar to ATCC 700518^T^ and SM1926 currently (Figure 3). Simultaneously, *P. arctica* A 37-1-1^T^ is in the deep branch between the *P. distincta* strains, clustering 21 genomes of the different closely related *Pseudoalteromonas* species based on the presence/absence patterns of 10,045 pan-genomic clusters (Figure 4). This bacterial intraspecific diversity may be mediated by such adaptive strategies as multiple nucleotide substitutions through random homologous recombination and mutations, multiplicity of essential genes, genome size and GC content regulation, and codon usage bias, where the members of a panmictic bacterial population co-occur or compete depending on prevailing conditions [38]. However, the acquisition of niche-adaptive genes and alleles may enable one recipient cell to invade a new niche, undergo clonal expansion, build a recombination barrier against genetic materials from the parental population, and eventually form a new species. The panmictic marine ancestral populations have been recently shown to undergo multiple speciation events in just tens of thousands of years [39]. The newly speciated populations, particularly those that have become symbiotic or have undergone other forms of local isolation, exhibit strong genetic isolation from one another, indicating a strict barrier to recombination. The rapid evolution of bacteria in the marine environment results in genome-wide phylogenetic incongruence among genes, sites within genes, and gene cluster rearrangements within genomes in newly divided species [39,40].

### 2.4. Phylogenetic Core-Genes Analysis of the P. distincta Strains

**Figure 5:**
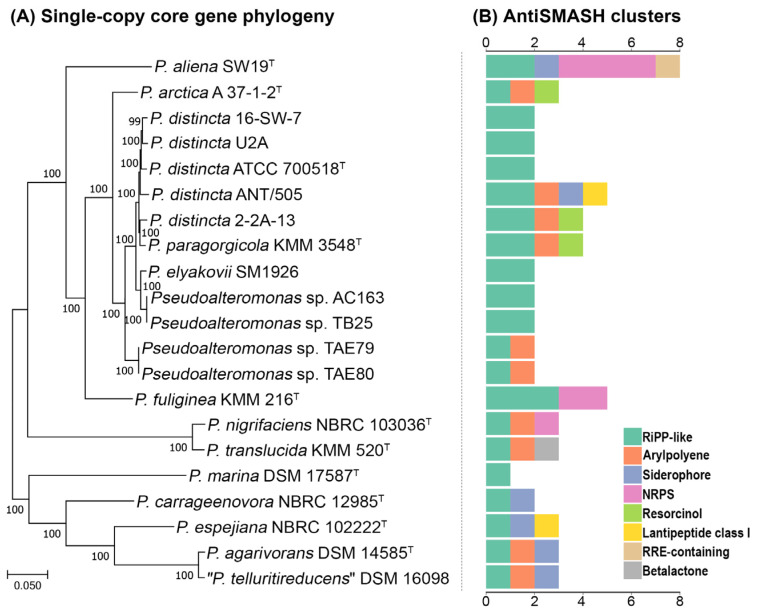
(**A**) Maximum-likelihood phylogeny of twenty-one *Pseudoalteromonas* strains based on 1369 core genes selected by Roary and reconstructed by IQ-TREE and (**B**) distribution of secondary metabolism biosynthetic gene clusters (BGCs) across the genomes.

The phylogenetic analysis based on the sequences of single-copy core genes was additionally performed to explore the phylogenetic relationships of *P. distincta* strains with the closely related species within the genus *Pseudoalteromonas* (Figure 5A). A core genome phylogeny using the concatenated sequences of 1369 core single-copy genes (1,320,659 base pairs, bp) showed that all *P. distincta* strains form a separate subclade with *P. arctica* A 37-1-1^T^ as the most closely related species. Moreover, three subclades could be distinguished, supported by the high bootstrap values. One subclade included the strain *P. distincta* ATCC 700518^T^ and the strains 16-SW-7, U2A, ANT/505, 2-2A-13, and KMM 3548. The second subclade included the strains SM1926, AC163, and TB25. The third subclade included the strains TAE79 and TAE80 (Figure 5A).

It has been shown earlier that the representatives of the genus *Pseudoalteromonas* are able to produce a broad array of bioactive molecules, among which are antibiotics, toxins/antitoxins, antitumor agents, and wide-spectrum enzymes with high specificities at low temperatures [4]. In general, the *Pseudoalteromonas* genus can be divided into the pigmented and non-pigmented species clades, where the pigmentation correlates with their proclivity for natural product formation. Conversely, the non-pigmented species of *Pseudoalteromonas* tend to possess unusual and diverse enzymatic activities (e.g., carragenases, chitinases, and alginases), generally broader environmental tolerance ranges (temperature, water activity, and pH), and substantially greater nutritional versatility compared to the pigmented species [6]. This could indicate that *Pseudoalteromonas* species members’ functions are narrowing to the host-associated lifestyle after they have evolved sufficient virulence factors as antioxidative carotenoid-like pigments to colonize the host in either a pathogenic or symbiotic manner [21,22,23,24,25].

### 2.5. Secondary Metabolite Biosynthetic Gene Clusters Analysis of the P. distincta Strains

**Figure 6:**
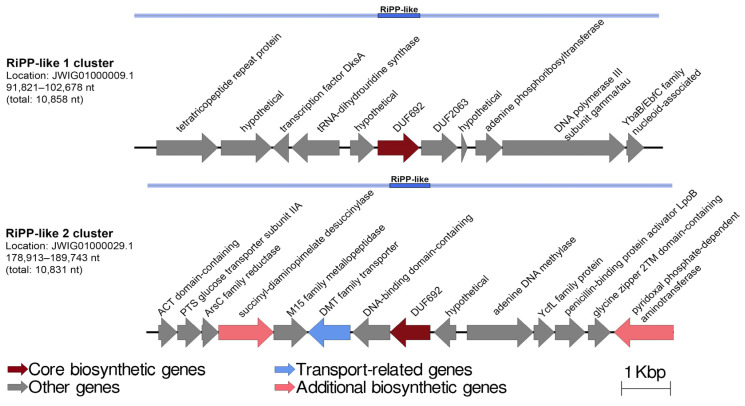
Putative biosynthetic gene clusters for RiPP-like peptides (1 cluster: nucleocidin-like; 2 cluster: brasilicardin-like) in the strains *P. distincta* and *P. arctica* A 37-1-2^T^.

The annotation of secondary metabolite biosynthetic gene clusters (BGCs) for 21 *Pseudoalteromonas* genomes revealed a lot of biosynthetic operons, namely: ribosomally synthesized and post-translationally modified peptides (RiPP)-like, arylpolyene (Polyketide synthase type II; PK), nonribosomal peptide-synthetase (NRPS), siderophore, resorcinol (hybrid PK/NRPS), lantipeptide class I, RiPP recognition element (RRE-containing), and betalactone. These results are also in agreement with Bosi et al. for the *Pseudoalteromonas* genus [4]. Ribosomally synthesized and post-translationally modified peptides (RiPPs) are ubiquitous among representatives of the genus *Pseudoalteromonas* and account for one to three biosynthetic clusters per genome (Figure 5B). In general, the RiPP clusters in *P. distincta* and *P. arctica* A 37-1-2^T^ (Figure 6) showed an identity with the pathway for flavin-dependent synthesis of bioactive adenosines (non-fluoro, 4′-fluoro-3′-*O*-β-glucosylated adenosine, and *O*-5′-sulfamyladenosine) produced by a soil bacterium *Streptomyces calvus* [41]. Other found RiPP clusters in *P. distincta* and *P. arctica* were slightly similar to the BGC from a soil-derived human pathogen, *Nocardia terpenica*, encoding for the known immunosuppressive metabolite, brasilicardin A, consisting of an unusual anti/syn/anti-perhydrophenanthrene skeleton with a carbohydrate side chain and an amino acid moiety [42].

**Figure 7:**
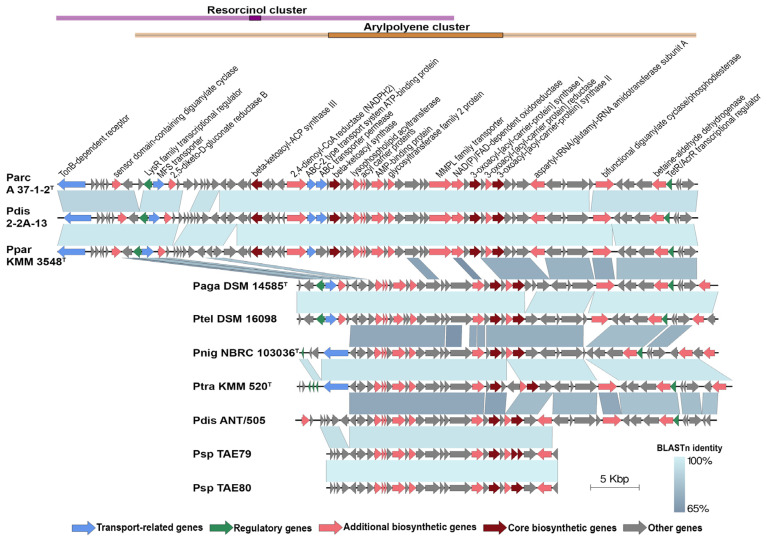
Comparative analysis of synteny between resorcinol and/or aryl polyene biosynthetic gene clusters (*ape* BGC), which were found in ten *Pseudoalteromonas* genomes: Parc A 37-1-2^T^, *P. arctica* A 37-1-2^T^; Pdis 2-2A-13, *P. distincta* 2-2A-13; Ppar KMM 3548^T^, *P. paragorgicola* KMM 3548^T^; Paga DSM 14585^T^, *P. agarivorans* DSM 14585^T^; Ptel DSM 16098, “*P. telluritireducens*” DSM 16098; Pnig NBRC 103036^T^, *P. nigrifaciens* NBRC 103036^T^; Ptra KMM 520^T^, *P. translucida* KMM 520^T^; Pdis ANT/505, *P. distincta* ANT/505; Psp TAE79, *Pseudoalteromonas* sp. TAE79; Psp TAE80, *Pseudoalteromonas* sp. TAE80. Genes were colored based on their annotations, as indicated at the bottom of the figure.

Another interesting cluster in *P. distincta* and *P. arctica* A 37-1-2^T^ is the pigment aryl polyene biosynthetic gene cluster (*ape* BGC). The bacterial pigments of the aryl polyene type are structurally similar to the well-known carotenoids with respect to their polyene systems. It has been demonstrated that carotenoids can affect membrane fluidity, and aryl polyene type pigment, like carotenoids, protect bacteria from reactive oxygen species [21,42,43]. Among the 21 studied genomes, aryl polyene biosynthetic gene clusters were found in ten genomes, including *P. arctica* A 37-1-1^T^, *P. distincta* ANT/505, 2-2A-13, *P. paragorgicola* KMM 3548, *Pseudoalteromonas* spp. TAE79 and TAE80, *P. nigrifaciens* NBRC 103036^T^, *P. translucida* KMM 520^T^, *P. agarivorans* DSM 14585^T^, and “*P. telluritireducens*” DSM 16098. The comparative analysis of synteny between the *ape* BGCs is presented in Figure 7.

As presented on the synteny plot, there are variations in cluster length and gene composition, especially in the number of core biosynthetic genes. In particular, the hybrid cluster PK/NRPS, containing the resorcinol BGC, has been found only in three strains: *P. distincta* 2-2A-13, KMM 3548 (formerly *P. paragorgicola* KMM 3548), and *P. arctica* A 37-1-1^T^, which coincide with their yellow-orange pigmentation phenotypes (Table 1, Figure 7). The microbial secondary metabolites, 2,5-dialkylresorcinols (DARs), which are derived from a condensation of two fatty acid metabolism intermediates, may be esterified with a non-isoprenoid aryl-polyene carboxylic acid in a flexirubin- or xanthomonadin-like manner [22,44]. The DARs’ bioactivities are known to be free radical scavengers and cell growth stimulating factors. Flexirubins are the DAR-*ape* orange pigments used as a chemotaxonomic marker for the environmental and gut bacteria of the *Bacteroidetes* phylum [44]. Thus, *P. arctica* A 37-1-1^T^ has slightly orange colonies on agar medium [32], and the production of the cell-bound yellow-orange pigments is also shown for the strains *P. distincta* 2-2A-13 and KMM 3548, which may indicate that their pigments are of the DAR-*ape* nature (Figure 1 and Figure 7). The higher level of the aryl polyene cluster identity of *P. distincta* and *P. arctica* was revealed with the *ape* BGC of an entomopathogenic symbiotic bacterium *Xenorhabdus doucetiae* from a nematode’s intestine [45]. The *ape* BGC synthesizes aryl-polyene lipids that protect the bacteria from oxidative stress and promote biofilm formation. It has been shown that the most widespread NRPS and hybrid PK/NRPS in *X. doucetiae* suppress insect immunity, allowing its host nematode to invade the insects, while the lactones are used by the bacterium against its soil microbial competitors [45].

### 2.6. Carbohydrate-Active Enzymes of the P. distincta Strains

**Figure 8:**
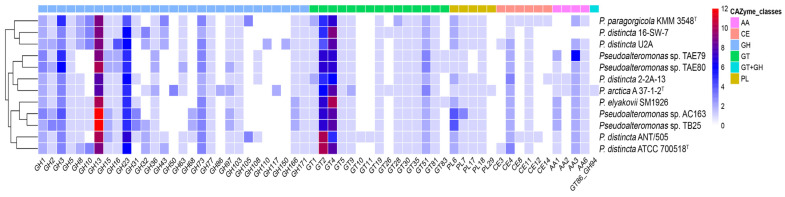
The distribution of carbohydrate-active enzymes (CAZymes) in the genomes of *P. distincta* and *P. arctica* A 37-1-2^T^. The heat map shows the number of genes assigned to individual CAZyme families. Rows are clustered using Euclidean distances. Parc A 37-1-2^T^, *P. arctica* A 37-1-2^T^; Pdis 2-2A-13, *P. distincta* 2-2A-13; Ppar KMM 3548^T^, *P. paragorgicola* KMM 3548^T^; Paga DSM 14585^T^, *P. agarivorans* DSM 14585^T^; Ptel DSM 16098, “*P. telluritireducens*” DSM 16098; Pnig NBRC 103036^T^, *P. nigrifaciens* NBRC 103036^T^; Ptra KMM 520^T^, *P. translucida* KMM 520^T^; Pdis ANT/505, *P. distincta* ANT/505; Psp TAE79, *Pseudoalteromonas* sp. TAE79; Psp TAE80, *Pseudoalteromonas* sp. TAE80.

Carbohydrate-Active enzymes (CAZymes) are the enzymes that synthesize, modify, or break down saccharides, and their classification comprises the following classes: glycoside hydrolase families (GHs), glycosyltransferase families (GTs), polysaccharide lyase families (PLs), carbohydrate esterase families (CEs), auxiliary activity families (AAs), and carbohydrate-binding module (CBM) families [46]. The enzymes from the *Pseudoalteromonas* strains, mainly cold-adapted as biological catalysts, could be applied in several industrial fields, spanning from the production of biofuels and food processing to applications in biological detergents and the paper industry, biodegradation of xenobiotic compounds in cold climates, and molecular and structural biology [6]. The distribution of CAZymes classes encoded in the genomes is shown in Figure 8.

We found that *Pseudoalteromonas* sp. AC163 possesses the highest number of CAZymes (90), followed by *Pseudoalteromonas* sp. TB25 (89), *P. distincta* ANT/505 (88), *P. distincta* ATCC 700518^T^ (85), *P. distincta* U2A (85), *P. paragorgicola* KMM 3548 (83), *Pseudoalteromonas* sp. TAE79 (81), *Pseudoalteromonas* sp. TAE80 (81), *P. elyakovii* SM1926 (78), and *P. distincta* 16-SW-7 (76). The smallest numbers of CAZymes, verified by the dbCAN2 databases, were in *P. distincta* 2-2A-13 (72). A total of 29 GH, 15 GT, 5 PL, 6 CE, and 4 AA families were classified in eleven *P. distincta* genomes. The most dominant were the GH13, GH23, GH3, GT4, GT2, PL6, CE4, and AA3 families in order of abundance. Based on the CAZy database (http://www.cazy.org/, accessed on 10 November 2022) definitions, the enzymes of predicted families might likely act as broad-spectrum glycosidases acting on substrates with α-glucoside linkages, *α*-amylases, peptidoglycan lyases, chitinases, *β*-D-glucosidases, *β*-D-xylosidases, *α*-L-arabinofuranosidases, *β*-*N*-acetyl-D-glucosaminidases, and alginate lyases. The GT2 and GT4 families were ranked as key glycosyltransferases, which are polyspecific enzymes. The CE family of four esterases catalyzes the deacylation of polysaccharides, and AA3 is widespread and catalyzes the oxidation of alcohols or carbohydrates with the concomitant formation of hydrogen peroxide or hydroquinones. These abundant enzymatic complexes in *P. distincta* and *P. arctica* may indicate the presence of active glycosylation and degradation processes in these strains (Figure 8).

However, the strains *P. distincta* ATCC 700518^T^, KMM 3548, 16-SW-7, and U2A are enriched in endo-1,4-β-xylanases and β-xylosidases of the families GH1, 3, 5, 8, 10, and 43 [47], suggesting an efficient utilization of hemicellulose in plants and algae, while the GHs 8, 10, and 43 are absent in the strains TAE79 and 80 (Figure 8). The strain *P. distincta* U2A is expectedly equipped with additional hydrolases capable of algal poly- and oligosaccharide degradation, such as GH110 (alpha-galactosidase) and GH150 (Ι-carrageenase) [5]. Although all *P. distincta* and *P. arctica* A 37-1-2^T^ strains were unable to degrade agar, the dbCAN discovered GH50 agarase in *P. arctica* A 37-1-2^T^ (Figure 8).

Part of the *P. distincta* strains, such as 2-2A-13, ANT/505, TAE79, and TAE80, have the genes encoding for the GH108 family responsible for hydrolysis of (1 → 4)-β-linkages between *N*-acetylmuramic acid and *N*-acetyl-D-glucosamine residues in peptidoglycans and between *N*-acetyl-D-glucosamine residues in chitodextrins (Figure 8). This could be due to their lysozyme antimicrobial activity (particularly against Gram-positive bacteria and fungi), which is commonly used for food safety (fruits, vegetables, meat, milk, and dairy products spoilage) due to its low toxicity in humans [48].

There is a structural family GH105, unsaturated rhamnogalacturonyl hydrolase or D-4,5-unsaturated -glucuronyl, galacturonyl hydrolases, with substrate specificity towards rhamnogalacturonan-I, ulvan, and arabinogalactan decorating certain cell wall proteins, especially planta glycans, in the strains KMM 3548 and ANT/505. This activity is intrinsic to the gut microbiota, particularly bacteria from the *Bacteroidetes* phylum [49].

### 2.7. Unique Genes and Genomic Islands Analysis of the P. disticta Strains

**Figure 9:**
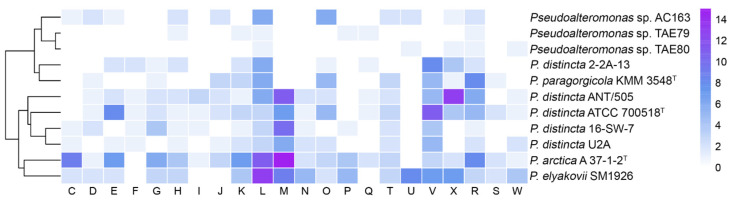
Functional annotation of unique genes among strains of *P. distincta* and *P. arctica* A 37-1-1^T^ (the *P. distincta* TB25 orthologues were mostly not found): Genes were assigned to the following COG categories: C, energy production and conversion; D, cell cycle control and mitosis; E, amino acid metabolism and transport; F, nucleotide metabolism and transport; G, carbohydrate metabolism and transport; H, coenzyme metabolism, I, lipid metabolism; J, translation; K, transcription; L, replication and repair; M, cell wall/membrane/envelope biogenesis; N, cell motility; O, post-translational modification, protein turnover, chaperone functions; P, inorganic ion transport and metabolism; Q, secondary structure; T, signal transduction; U, intracellular trafficking and secretion; V, defense mechanisms; X, mobilome: prophages, transposons; R, general functional prediction only; S, function unknown; W, extracellular structures.

The variable part of the pan-genome relates to the accessory or unique features, common to several or limited to individual strains, respectively, and associates with metabolic variability and ecological differentiation within the species or population, resulting in “microdiversity” of phenotypic traits [40,50,51]. It is interesting that each of the *P. distincta* and *P. arctica* A 37-1-1^T^ genomes contain from 7 to 210 unique genes, the orthologues of which are absent in other *Pseudoalteromonas* genomes (Figure 4 and Figure 9).

The largest number of unique genes was observed in the genomes of *P. distincta* SM1926 (=*P. elyakovii SM1926*, NCBI) (210 genes), *P. distincta* ANT/505 (196), *P. arctica* A 37-1-1^T^ (195), *Pseudoalteromonas* sp. AC163 (142), and *P. distincta* ATCC 700518^T^ (107) in order of abundance (Figure 9). The genomes of *P. distincta* 2-2A-13, *P. paragorgicola* KMM 3548, *P. distincta* 16-SW-7, *P. distincta* U2A, *Pseudoalteromonas* sp. TAE79, *Pseudoalteromonas* sp. TAE80, and *Pseudoalteromonas* sp. TB25 account for 97, 97, 94, 66, 15, 11, and 7 unique genes, respectively. According to the COG class annotation of these unique genes, the most abundant functional classes were replication, recombination, and repair (13.59% of total unique gene clusters), defense mechanisms (13.59%), cell wall/membrane/envelope biogenesis (12.99%), general function prediction only (8.15%), mobilome: prophages, transposons (6.94%), posttranslational modification, protein turnover, chaperones (6.04%), transcription (5.13%), amino acid transport and metabolism (4.83%), carbohydrate transport and metabolism (3.93), and signal transduction mechanisms (3.92%). Apparently, these genes might be responsible for the functional differences between the strains of *P. distincta*. In general, the analysis of the pangenome, including selected genomes of the genus *Pseudoalteromonas*, revealed a high percentage of unique genes that agrees with the data observed by Bosi et al. (2017) [4]. Remarkably, the *P. distincta* genes are strongly strain-specific, with the exception of seven common genes for *P. distincta* strains and *P. arctica* A 37-1-2^T^ (Supplementary Table S5).

The variable genetic repertoire is often encoded in equivalent loci in different strains, genomic islands (GI), with an active mechanism of genetic exchange [50,52,53]. They are known to influence niche specialization in cyanobacteria, actinobacteria, roseobacteria, and marine gamma proteobacteria by regulating carbon metabolism, production of siderophores, bacteriocins, and flagellar assembly [39,50].

In the GIs of eleven *P. distincta* strains and one *P. arctica* A 37-1-1^T^, 587 genes were identified, most of which belong to transposase families (60 genes), translocases (6), transcriptional regulators (39), phosphate metabolism (40), DNA-binding and modifying proteins (24), transporters (23 genes, including B12, riboflavin, glycine betaine, malate), methyltransferases (19), multidrug and metal resistance (12), sulfate metabolism (11), 50S ribosomal proteins (9), sensors (6), flagella-associated proteins (6), secretion systems of type I, II and III (6), ferredoxin-like proteins (5), quinone reductases (5), permeases (5 genes, including xanthine, uracil, raffinose, phosphate), integrases (4), exporters (5 genes, including lipopolysaccharides, threonine/homoserine, copper), tyrosine and serine recombinases for site-specific recombination (3), nucleases and nucleotid-association proteins (20), proteases (10), and shock proteins (4—cold and heat shock, and phage shock (only in the free-living strain KMM701) (Supplementary Table S6). Many additional genes were from lipid and fatty acid metabolism (phosphatidylethanolamine transferase, phospholipase, lipoprotein transferases, diacylglycerol kinase, etc.). Notably, 16 biosynthetic genes were related to RNA and tRNA pseudouridin, GMP, glycans, riboflavin, folate, and terpene production. Twenty-one genes were dehydrogenases from lactate, glutarate, xanthine, alcohol, phosphonate, threonine, aspartate, erythronate, long-chain aldehyde, acetaldehyde, glycerol and glyceraldehyde phosphates, glycine, shikimate, glutathione, aldose sugar, D-beta-hydroxybutyrate, and inositol metabolism pathways. Twelve gene annotations predicted hydrolytic functions towards S-formylglutathione, hydroxyacylglutathione, oxamate, ADP, deoxyuridin-3-phosphate, and 5-hydroxyisourate (Supplementary Table S6).

Exclusively, two carrageenases, one ulvan-active sulfatase, and four *α*-1,3-galactosidases were found in the GIs of the strain *P. distincta* U2A, found in a brown alga *Fucus* sp., and consequently adapted to fermentation of the algal polysaccharides (Supplementary Table S6). Export of lipopolysaccharides and adaptive-response sensory kinase SasA, responsible for binding to the innate immune receptor glycoprotein during bacterial colonization of a host epithelial tissue, were found only in the strain ATCC 700518^T^, which has been suggested to become a sponge-associated pathogen [1,54]. Although the *P. distincta* strains did not hydrolyze urea (Table 1), such virulent factors as urease (subunits alpha and beta) and its accessory proteins (a cluster of 7 genes) are also located only within the GIs of *P. distincta* ATCC 700518^T^, indicating the acquired nature of its ability to assimilate urine intensively [1]. However, the urease genes could be transferred from cyanobacteria, which are currently being tested as a way to recycle wastes, CO2, and urea [55]. Notably, peptidoglycan-associated lipoproteins occurred in all *P. distincta* host-associated isolates (Supplementary Table S6), but UDP-2,4-diacetamido-2,4, 6-trideoxy-beta-L-altropyranose hydrolase, which is involved in biosynthesis of pseudaminic acid as a component of polysaccharides in certain gut pathogens, occurred in the strains KMM 3548 and 2-2A-13 with carotenoid-like phenotypes [56]. Some other genes related to the UDP sugar metabolism and polysaccharide synthesis, including the sialic-acid-like sugar pseudaminic acid, which is used to modify flagellin, were present in the GIs of carotenoid-like strains [57]. The putative cold-specific marine lipooligosaccharide in strain 2-2A-13, produced with participation of 8-amino-3,8-dideoxy-manno-octulosonate cytidylyltransferase (Supplementary Table S6), was first discovered in *Shewanella* sp. HM13, isolated from a horse mackerel’s intestine [58].

The enzyme 3-aminobutyryl-CoA aminotransferase was also presented only in the carotenoid-type strains KMM 3548 and 2-2A-13, which are suggested to be used in an alternative lysin fermentation pathway in the digestive tract of many organisms [59]. In general, there are several additional genes of this biosynthetic pathway (DAP) in the GIs leading to conversion from aspartate to lysine in the GIs of many *P. distincta* strains (diaminopimelate epimerase, diaminopimelate decarboxylase, and *N*-acetyldiaminopimelate deacetylase) (Supplementary Table S6).

Remarkably, no genes for carotenoids, aryl polyenes, or other pigment synthesis pathways were found in the GIs expected for homogentisate 1,2-dioxygenase in the strain AC163 from the arctic marine sponge, which may be involved in the catabolism of phenylalanine and tyrosine leading to melanin pigment formation [8,26].

### 2.8. Metabolic Pathways Analysis of the P. distincta Strains

**Figure 10:**
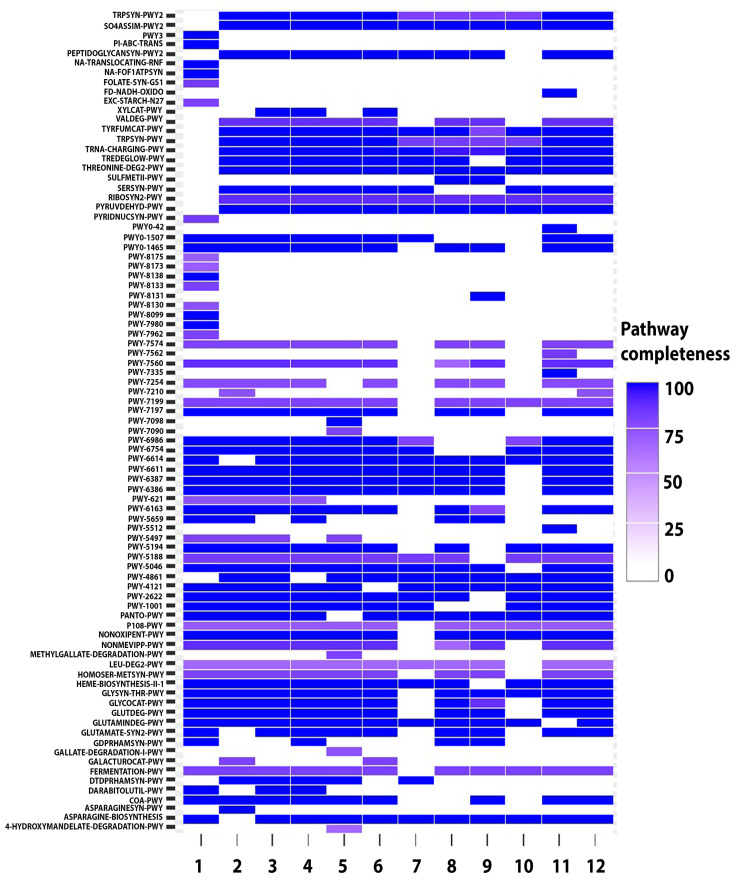
Strain-specific metabolic pathways in *P. distincta* and *P. arctica* A 37-1-1^T^ (pathway signatures are depicted in the corresponding Supplementary Table S5): 1—*P. distincta* 16-SW-7 (GCA_005877035.1; seawater); 2—*P. distincta* ANT 505 (GCA_000212655.3; seawater); 3—*P. distincta* ATCC 700518^T^ (GCA_000814675.1; marine sponge); 4—*P. distincta* U2A (GCA_008370225.1; brown algae surface); 5—*P. elyakovii* SM1926 (GCA_007786285.1; surface seawater); 6—*P. paragorgicola* KMM3548 (GCA_014918315.1); 7—*Pseudoalteromonas* sp. AC163 (GCA_000497935.1; marine sponge); 8—*Pseudoalteromonas* sp. TAE79 (GCA_000498015.1; water column); 9—*Pseudoalteromonas* sp. TAE80 (GCA_000498035.1; water column); 10—*Pseudoalteromonas* sp. TB25 (GCA_000497995.1; Antarctic marine sponge); 11—*P. arctica* A 37 1 2T (GCA_000238395.4; seawater); 12—*P. distincta* 2-2A-13.

The genes for carotenoid and unique metabolism pathways were being searched in eleven *P. distincta* and one *P. arctica* A 37-1-1^T^ strains by the metabolic reconstruction implemented by gapsec, a novel powerful tool for bacterial phenotype prediction from the genotype (Figure 10, Supplementary Tables S7 and S8). Currently, gapseq outperforms state-of-the-art tools in predicting enzyme activity, carbon source utilization, fermentation products, and metabolic interactions within microbial communities, as proved by the experimental data for 14,931 bacterial phenotypes [20].

81 and 86 metabolic pathways, with a completeness of 66 to 100%, were estimated in the *P. distincta* and *P. arctica* A 37-1-1^T^ strains as true identical and true different, respectively (Figure 10; Supplementary Tables S7 and S8). These bacteria use glycolysis III, gluconeogenesis, the glyoxylate cycle, 2-oxoglutarate decarboxylation to succinyl-CoA (the TCA pathway), and fatty acid beta-oxidation pathways. The glyoxylate shunt for marine heterotrophic bacteria has been shown to be an important adaptation strategy in the presence of Fe-limitation [60]. In addition to the abundant amino acids, nucleic acids, and cofactor metabolism pathways (Supplementary Tables S7 and S8), the ability of *P. distincta* and *P. arctica* to degrade fatty acids through the fatty acid beta-oxidation and superoxide radical degradation pathways provides their competitive advantage to adapt to different niches and environments [61]. Although all strains are strictly aerobes (Table 1), they have some common traits of the soil saprophytes, endogenous plant or intestinal bacterial communities, such as B12-dependent ethanolamine utilization, 3-methylthiopropanoate biosynthesis, pyruvate fermentation to propanoate I, ethene biosynthesis III, acetate and ATP formation from acetyl-CoA II, cyanide degradation, sulfoacetaldehyde degradation I, formaldehyde oxidation II (glutathione-dependent), choline degradation II, glycine betaine biosynthesis I, folate polyglutamylation, polyamines biosynthesis, sucrose degradation IV (sucrose phosphorylase), phosphate acquisition, assimilatory sulfate reduction, and aromatic compound catabolism (Supplementary Tables S7 and S8). However, the same several pathways have been described in halophilic archaea and marine bacteria [62,63].

Remarkably, all strains of *P. distincta* and *P. arctica* A 37-1-1^T^ have 88% completeness of the common pathway for streporubin B synthesis (Supplementary Tables S7 and S9) related to prodiginines, a family of red-pigmented tripyrrole antibiotics that destroy DNA, found in *Actinomycetes* and other eubacteria and associated with significant antitumor, immunosuppressive, anti-inflammatory, anti-malarial, anthelmintic, antifungal, and antibacterial activities. This highly toxic metabolite has been suggested to trigger programmed cell death and morphological changes from mycelia to sporulation in *Streptomyces* sp. [64].

As for the different metabolic properties of *P. distincta* and *P. arctica* A 37-1-1^T^, the polysaccharide metabolism was much more strain-specifically variable (Figure 10, Supplementary Table S8). The variations in sugar content of the O-antigens contribute to the wide variety of antigenic types between the species and strains: UDP-*N*-acetyl-alpha;-D-mannosaminouronate biosynthesis in *P. arctica*, UDP-alpha-D-galacturonate biosynthesis I (from UDP-D-glucuronate) in *P. distincta*, with the exception of 16-SW-7 and U2A. The nucleotide-activated donor of D-rhamnose units to the A-band lipopolysaccharide was absent only in TB5. Whereas, GDP-mannose biosynthesis, as well as succinoglycan biosynthesis, were found exclusively in the free-living strain 16-SW-7 (Figure 10; Supplementary Table S8). Bacterial succinoglycan is found suitable as a viscosifying and emulsifying agent in the food industry, in gravel packing, or as a fluid loss control agent [28]. UDP-*N*-acetylmuramoyl-pentapeptide (lysine- and meso-diaminopimelate-containing) biosynthesis II for building the cell wall peptidoglycans was absent only in TB25, while UDP-*N*-acetyl-D-galactosamine biosynthesis I (mucin-like glycoprotein) was only in *P. arctica.* The pathway of dTDP-beta-L-rhamnose biosynthesis occurred in the *P. distincta* strains U2A, SM1926, and AC163. UDP-beta-L-arabinose biosynthesis I (from UDP-alpha-D-xylose), UDP-alpha-D-glucuronate biosynthesis (from UDP-glucose), CMP-3-deoxy-D-manno-octulosonate biosynthesis, and cellulose and trehalose biosynthesis IV signatures were found in almost all strains (Figure 10; Supplementary Tables S7 and S8). The nucleotide sugars, activated forms of monosaccharides, are required substrates for the corresponding glycosyltransferase-catalyzed synthesis of polysaccharides, which comprise important biopolymers in all living organisms, such as glycosylation of signaling receptors, core protein linkage formation in proteoglycans, synthesis and rearrangement of glycoproteins and lipopolysaccharides in the cell walls, and promotion of the viability and virulence of many pathogens [57,65]. That is why de novo pathways for nucleotide sugar biosynthesis may be biotechnology relevant for use in drug development, cosmetology, and industry [30,66].

The sugar and polysaccharide degradation pathways, as well as amino acids, vitamins, and cofactor metabolism, also significantly differed in the *P. distincta* and *P. arctica* A 37-1-1^T^ strains (Figure 10; Supplementary Table S8). Although not many genes of the family PL with a high identity have been shown for *P. distincta*, the alginate degradation pathway is rather an intrinsic property for the strains studied, except for *P. distincta* TAE79 and TAE80 (Figure 8). Exclusively, the pathway of 3,6-anhydro-alpha-L-galactopyranose degradation was in the strain *P. arctica* A 37-1-1^T^, related to utilization of the red macroalgal polysaccharides agarose and porphyran as renewable biomass for biofuel and chemical production [29]. Unexpectedly, the putatively free-living strain *P. distincta* 16-SW-7 has the highest numbers of additional pathways relative to other strains (Figure 10; Supplementary Table S8). In the strain 16-SW-7, the pathways of pyruvate decarboxylation to acetyl CoA I, flavin biosynthesis I, L-serine biosynthesis I, L-threonine degradation II, trehalose degradation I (low osmolarity), L-tryptophan biosynthesis, L-tyrosine degradation I, and L-valine degradation I were absent; while 19 additional pathways were presented, including extracellular starch(27n), D-arabitol and sucrose degradation III (sucrose invertase), GDP-mannose and succinoglycan biosynthesis, 4-hydroxymandelate degradation (aromatic compounds metabolism), polyphosphate metabolism, anteiso-branched- and even iso-branched-chain fatty acid biosynthesis (maintain membrane fluidity through “homeoviscous adaptation”), ATP and NAD de novo (from aspartate) biosynthesis I, 5′-deoxyadenosine degradation I, leucyl aminopeptidase test (UV tolerance in cyanobacteria), Na()-translocating ATPase and ferredoxin:NAD() oxidoreductase complex, and tetrahydropteridine recycling (Figure 10; Supplementary Table S8).

In addition, the strain *P. distincta* 16-SW-7 seems to be able to synthesize adenosylcobinamide-GDP from cobyrinate *a*,*c*-diamide at the late steps in the biosynthesis of vitamin B12, where the early-insertion and late-insertion pathways combine. In this pathway, the lower ligand base of cobamides is tethered to the corrin ring via the nucleotide loop, which is composed of (R)-1-amino-2-propanol O-2-phosphate and 5,6-dimethylbenzimidazole [67]. The additional pathways for aerobic aromatic compound catabolism —the degradation of methylgallate and gallate (II), vanillin and vanillate (II), whose major sources are plant lignin and tannins—were found in the strain *P. distincta* SM1926 (Figure 10; Supplementary Table S8). All of these traits may be associated with high environmental durability and more virulence factors [68].

The L-tyrosine degradation pathway (I) may lead to melanin production in *P. distincta* (eumelanin or pyomelanin), with the exception of the non-pigmented strain *P. distincta* 16-SW-7 [26]. Tetrahydrobiopterin, which was only in *P. distincta* 16-SW-7, has been confirmed to inhibit uncompetitively tyrosinase due to specific binding of the pyrimidine ring of the pterin moiety to its regulatory domain; under these conditions, there was no reduction of L-dopaquinone back to l-DOPA [69]. However, the strain 16-SW-7 may compensate for the absence of antioxidative melanin or carotenoid-like pigmentation (Table 1) by tetrahydropteridine recycling due to its feasibility of auto- and photosensitized oxidation in the presence of singlet (^1^O_2_) and molecular (^3^O_2_) oxygen [70].

Nevertheless, inspecting the genes belonging to carotenoid synthesis/degradation pathways, the signatures identified by gapsec for all strains of *P. distincta* and *P. arctica* A 37-1-1^T^ were the following: nonmevanolate isoprenoid methylerythritol phosphate (MEP) pathway II; geranyl diphosphate biosynthesis; and trans, trans-farnesyl diphosphate biosynthesis, with a completeness of 88, 100, and 66, respectively, excepting for the strains AC163 and TB25 (Supplementary Tables S7 and S9). Among the putative carotenoid-like pathways, which were manually verified by blasting their gene candidates, the biosynthesis of 4,4′-diapolycopenedioate and/or staphyloxanthin biosynthesis of (2E,6E)-farnesyl diphosphate (C15-isoprenoid) are most probable in *P. distincta* and *P. arctica* A 37-1-1^T^ due to the good blast results for key genes: 4,4′-diapolycopenedial and 4,4′-diaponeurosporenal synthases/dehydrogenases, respectively (Supplementary Tables S8 and S9). These enzymes have been shown to be involved in the biosynthesis of relatively rare C30 carotenoids (triterpenoids), such as yellow-orange staphyloxanthin from *Staphylococcus aureus*, which plays a role in virulence via detoxifying reactive oxygen species that are generated by a host’s innate immune system, and 4,4′-diapolycopene acid from methylotroph *Methylomonas* [13]. Both carotenoid pathways continue in a similar manner: activation of both ends of the molecule by diapolycopene oxygenase, forming aldehyde groups, followed by further oxygenation by 4,4′-diapolycopenedial dehydrogenases to form carboxylate groups. The last part of the pathway, further modification forming 4,4′-diapolycopenedioate glycosyl diester in *Methylomonas* has not been characterized yet, in contrast to the glycosyltransferase-mediated formation of the analogous glucosyl C30-carotenoid in the marine bacterium *Planococcus maritimus* with the orthologous gene cluster [10]. However, all genes encoding for the enzymes from the putative C30-carotenoid biosynthetic pathway in *P. distincta* and *P. arctica* have yet to be elucidated (Supplementary Tables S8 and S9).

### 2.9. Emended Description of the Species Pseudoalteromonas distincta (Romanenko et al., 1995; Ivanova et al., 2000; Nedashkovskaya et al., 2022)

The description of the species *Pseudoalteromonas distincta* is as given by Romanenko et al. [31], Ivanova et al. [3], and Nedashkovskaya et al. [1], with the following modifications and amendments. The temperature for growth ranges from 4 to 37◦C. In the API ID 32GN gallery, the majority of strains are positive for the assimilation of D-glucose, maltose, sucrose, D-mannitol, sodium acetate, sodium citrate, L-alanine, L-serine, L-proline, glycogen, propionic acid, valeric acid, and capric acids. Production of acetoin is strain-dependent. The genomic DNA G + C content is 39.1–39.3 mol%.

The type strain is KMM 638^T^ (=ATCC 700518^T^), isolated from a marine sponge collected at a depth of 350 m near the Komandorskie Islands, Russia. The GenBank/EMBL/DDBJ assembly accession number for the genome of this type of strain is GCA_000814675.1.

## 3. Material and Methods

### 3.1. Bacterial Strains for Phenotype Characterization

The strains under study were isolated from various marine environments (Table 1). The type strain of the species *Pseudoalteromonas* (*Alteromonas*) *distincta* KMM 638^T^ (=ATCC 700518^T^) was associated with a marine sponge collected near the Komandorskie Islands (Bering Sea, Pacific Ocean) at a depth of 350 m [31]. The strains *P. distincta* 16-SW-7 (=KMM 701) and *P. arctica* A 37-1-2^T^ were recovered from seawater samples collected near Island Paramushir (Kuril Islands, Okhotsk Sea, Pacific Ocean) and Spitsbergen Archipelago, respectively (Arctic Ocean) [1,32]. The strain *P. distincta* KMM 3548, formerly the type strain of the species *P. paragorgicola*, was isolated from the gorgonian *Paragorgia arborea*, collected near Island Onekotan (Kuril Islands, Okhotsk Sea, Pacific Ocean) at a depth of 202 m [1]. At last, the strain *P. distincta* 2-2A-13 (=KMM 6257) was isolated from the starfish *Leptasterias* sp., collected in the North Kuril Islands area at a depth of 584 m (Okhotsk Sea, Pacific Ocean) (this study). For morphological, biochemical, physiological, and chemotaxonomic characterization, the strains were grown under optimal physiological conditions for all strains (at 28 °C for 24 h on marine agar) as previously described [1]. Similar data for the strain *P. arctica* A 37-1-2^T^ can be found in [32].

### 3.2. Bacterial Strains for Genome Characterization

For genomic analysis, the genomes of the strains *P. distincta* KMM 3548 (GCF_014918315.1) and *P. arctica* A 37-1-2^T^ (GCA_000238395.4) were obtained from the GenBank, NCBI (Supplementary Table S2). The strains *P. distincta* ATCC 700518^T^ (GCF_000814675.1), *P. distincta* 16-SW-7 (GCF_005877035.1), *Pseudoalteromonas* sp. AC163 (GCF_000497935.1), *Pseudoalteromonas* sp. TB25 (GCF_000497995.1), *Pseudoalteromonas* sp. TAE79 (GCF_000498015.1), *Pseudoalteromonas* sp. TAE80 (GCF_000498035.1), *P. flavipulchra* ATCC BAA-314^T^ (GCF_000803085.1), *P. distincta* ANT/505 (GCF_000212655.2), *P. elyakovii* SM1926 (GCF_007786285.1), and *P. distincta* U2A (GCF_008370225.1) were taken as belonging to the species *P. distincta* based on the available results on the whole-genome sequences analysis, implemented by the Comparative Genomics (CG) on the server EzBioCloud (Supplementary Table S3).

For the species name validation, whole-genome sequencing was carried out for the strains *P. distincta* 2-2A-13 (=KMM 6257) and *P. elyakovii* VKPM B-3905^T^ (this study).

### 3.3. Whole-Genome Sequencing and Assembly

The genomic DNA was obtained from the bacterial cultures of the strains *P. distincta* 2-2A-13 and *P. elyakovii* VKPM B-3905^T^ using the NucleoSpin Microbial DNA Mini kit (Macherey-Nagel, Düren, Germany), following the manufacturer’s instructions. Whole-genome shotgun sequencing of the strains was carried out on an Illumina MiSeq platform using Nextera DNA Flex kits (Illumina, San Diego, CA, USA) and a 150-bp paired-end sequencing kit (Illumina, San Diego, CA, USA). The sequence quality was assessed via FastQC version 0.11.8 [71], and reads were trimmed using Trimmomatic version 0.38 [72]. Filtered reads were assembled de novo with SPAdes version 3.15.3 [73], and assembly metrics were calculated with QUAST version 5.0.2 [74]. The draft genomes of the strains were annotated using the NCBI Prokaryotic Genome Annotation Pipeline (PGAP) [75]. The genomes of the strains *P. distincta* 2-2A-13 and *P. elyakovii* VKPM B-3905^T^ were deposited in GenBank/EMBL/DDBJ under the accession numbers JANIHL010000000 and JANJFO010000000, respectively.

### 3.4. Whole-Genome, Core-Genome, and Pangenome Phylogeny

Representative genomes of the type strains of the type species for the genus *Pseudoalteromonas* were retrieved from GenBank, NCBI, using the ncbi-genome-download version 0.3.0 (https://github.com/kblin/ncbi-genome-download, accessed on 26 July 2022). The GenBank accession numbers for the genomes used in this study are listed in Supplementary Table S2. The phylogenetic analysis was performed with PhyloPhlAn version 3.0.1 using 400 conserved protein sequences, and a maximum-likelihood tree was reconstructed by RAxML version 8.2.12 under the LG + Γ model with non-parametric bootstrapping using 100 replicates [34,76]. The Average Nucleotide Identity (ANI) and Amino Acid Identity (AAI) values were calculated with the online server ANI/AAI-Matrix [77]. Values of in silico DNA–DNA hybridization (dDDH) of the studied strains and their closest relatives were measured at the TYGS platform (formula d4) [78].

Prokka version 1.14.6 was used for the annotation of the 21 genome sequences of the *P. distincta* strains and their phylogenetic relatives, with default parameters [79]. The obtained GFF files were used for core gene identification using Roary version 3.13.0 with the following flags: -e, -n, and -i [80,81]. A core genome phylogeny based on concatenated 1369 core gene sequences (composite length of 1,320,659 bp) was reconstructed with IQ-TREE version 2.2.0.3 and ModelFinder under the GTR + F + I + I + R8 model with non-parametric bootstrapping using 100 replicates [82,83].

The pan-genome for the 21 *Pseudoalteromonas* strains was reconstructed using the microbial pangenomics workflow in Anvi’o version 7.1 [84]. Annotated by Prokka, GFF files were imported into the Anvi’o contigs database using the script gff_parser.py (https://github.com/karkman/gff_parser, accessed on 5 October 2022). The genomes were organized based on the distribution of gene clusters using the MCL algorithm (distance: Euclidean; linkage: Ward).

Genome-wide analyses of orthologous clusters and pairwise genome comparisons of eleven *P. distincta* strains and one *P. arctica* A 37-1-2^T^ were performed using OrthoVenn2 (https://orthovenn2.bioinfotoolkits.net/home, accessed on 28 November 2022) [85].

### 3.5. Comparative Analysis of CAZy Families and Biosynthesis Gene Clusters

To identify carbohydrate-active enzymes (CAZymes), the dbCAN2 meta server version 10 was used with default settings (http://cys.bios.niu.edu/dbCAN2, accessed on 3 February 2022) [81,86]. The predictions by two of the three algorithms integrated within the server (DIAMOND, HMMER, and dbCAN-sub) were considered sufficient for CAZy family assignments. Annotation of secondary metabolite biosynthetic gene clusters was conducted using antiSMASH server version 6.1.1 (https://antismash.secondarymetabolites.org/#!/start, accessed on 25 September 2022). The relative abundances of CAZymes and antiSMASH clusters were visualized by heat maps and stacked bar plots using the heatmap version 1.0.12 and ggplot2 version 3.3.5 packages in RStudio version 2022.02.0+443 with R version 4.1.3. The genomic regions containing resorcinol and aryl polyene biosynthetic gene clusters were extracted from the GBK files of the genomes using Geneious Pro software version 4.8 [87]. The identifiers for genes included in the selected loci are listed in Supplementary Table S11. Generated GBK files were modified by adding custom color feature qualifiers, according to the antiSMASH conventional coloring. Pairwise comparisons of each locus between ten genomes were carried out using BLASTn (BLAST version 2.11.0+) run in EasyFig (version 2.2.5) [88]. Synteny plots were visualized by Easyfig with a minimum BLAST hit of 680 bp. Fonts and sizes in all figures were edited manually in Adobe Photoshop CC 2018 for improved visualization.

### 3.6. Genomic Island Analysis

Genomic island (GI) prediction was made using the IslandViewer 4 [52]. The alignments were performed against *P. distincta* 16-SW-7 (GCA_005877035.1) as a reference finished genome. IslandViewer integrates two sequencecomposition GI prediction methods, SIGI-HMM and IslandPath-DIMOB, and a single comparative GI prediction method, IslandPick. These methods have varying advantages and disadvantages. Predictions of virulence factor homologs for certain genomes are provided to indicate genes of potential interest but require further manual investigation of their role in virulence.

### 3.7. Reconstruction of Metabolic Pathways

Metabolic pathways for all studied strains were reconstructed using gapseq 1.2 [20]. The pathway prediction part of gapseq is implemented as a Bash shell script, and the metabolic model generation part is written in R. Linear optimization can be performed with different solvers (GLPK or CPLEX). Other requirements are exonerate, bedtools, and barrnap. In addition, the following R packages are needed: data.table [89], stringr [90], sybil [91], getopt [92], reshape2 [93], doParallel [94], foreach [95], R.utils [96], stringi [97], glpkAPI [98], and BioStrings [99]. Models can be exported as SBML [100] files using sybilSBML [91] or R data format (RDS) for further analysis in R, for example with sybil [91] or BacArena [86]. The PubChem [101] and BioCyc [102] databases were used to analyze the discovered metabolic pathways. The bash and R scripts were provided by https://github.com/OxanaKolpakova/metabolic-scripts (accessed on 30 January 2023).

## 4. Conclusions

Based on the comprehensive genome-based and phenotypic characterization, the strains ATCC 700518^T^ (=KMM 638T), 16-SW-7 (=KMM 701), KMM 3548, and 2-2A-13 (=KMM 6257), U2A, AC163, TB25, TAE79, TAE80, ANT/505, and SM1926 have been assigned to the species *P. distincta.* The description of the species is amended, particularly in the temperature growth range of 4–37◦C. The phenotype, chemotaxonomy, 16S rRNA sequences, metabolic pathways, secondary metabolite biosynthetic gene clusters, pangenome-specific genes, and protein sequences (94.70–95.51% ANI) are common for the strains *P. distincta* and *P. arctica* A 37-1-2^T^. However, the low DNA hybridization indexes dDDH (52.7–56.9%) indicate species delineation (≤70%). Although all strains possess putative methylerythritol phosphate (MEP) pathway II, 4,4′-diapolycopenedial biosynthesis enzymes (C30 carotenoids), and aryl polyene biosynthetic gene clusters (*ape* BGS), the carotenoid-like pigmentation traits in three strains, *P. distincta* KMM 3548, KMM 6257, and *P. arctica* A 37-1-2^T^, coincide with the presence of the hybrid cluster PK/NRPS, which encodes for the synthesis of aryl polyene esterified with resorcinol. Among the predicted biologically active compounds of *P. distincta* and *P. arctica* A 37-1-2^T^, the putative glycosylated immunosuppressants, similar to brasilicardin A, streptorubin B, and adenosine-containing nucleocidin, as well as a variety of sugar nucleotides and alginate derivatives, are more valuable secondary metabolites for further structural and biological studies. The starch, agar, carrageenan, xylose, lignin-derived, and other aromatic compound degradation pathways, as well as polysaccharide, folate, and cobalamin biosynthesis, are strain-specific for *P. distincta*. Each isolate is a significant contributor to the currently open pan-genome of the genus *Pseudoalteromonas*.

## Figures and Tables

**Table 1:** Phenotypic characteristics of strains belonging to the species *P. distincta* and *P. arctica* A 37-1-2^T^: Strains: 1, KMM 638^T^ [1,3,31]; 2, 16-SW-7 (=KMM 701) [1]; 3, KMM 3548 [1]; 4, 2-2A-13 (=KMM 6257; this study); 5, *P. arctica* A 37-1-2^T^ [32].

Characteristic *	1	2	3	4	5
Source of isolation	Sponge	Seawater	Gorgonian	Starfish	Seawater
Colony color	whitish	whitish	Slightly orange	Slightly orange	Slightly orange
Temperature range for growth (°C)	4–30	4–34	4–30	4–37	4–25
Salinity range for growth (% NaCl)	1–6	1–10	0.5–8	0.5–10	0–9
Required seawater or artificial seawater for growth	+	−	−	−	−
Production of melanin-like pigments	+	−	−	−	−
Acetoin production	−	−	−	w	−
H2S production	+	−	+	+	−
Hydrolysis of:					
Casein	−	w	w	+	+
Gelatin	+	+	+	+	−
Starch	−	−	+	+	−
Acid production from:					
Cellobiose, maltose	−	+	+	+	+
Galactose	−	+	+	−	+
Glucose, lactose, xylose	−	+	+	−	−
Raffinose	−	+	−	−	−
Mannitol	+	+	−	+	+
Utilization of citrate	−	+	w	w	+
Assimilation of:					
D-Glucose, sucrose, sodium acetate, L-alanine, L-serine, capric acid	+	+	+	−	+
Glycogen	+	+	+	−	−
Sodium citrate, L-proline, propionic acid, valeric acid	+	+	+	−	ND
Potashium-2-keto-gluconate, salicin	−	+	−	−	−
Itaconic acid, L-histidine	−	+	−	−	ND
D-Melibiose	−	+	+	−	+
D-Sorbitol	−	+	+	−	−
Inositol, lactic acid, ribose	+	−	−	−	−
Sodium malonate, 3-hydroxybutiric acid	+	−	−	−	ND
Enzyme activity (API ZYM tests):					
Esterase (C4)	+	+	−	+	ND
Cysteine arylamidase	−	+	−	w	ND
Trypsin, α-chymotrypsin	−	+	−	−	ND
β-Glucosidase, β-galactosidase	−	−	−	−	+
Susceptibility to:					
Ampicillin, vancomycin	+	−	+	+	ND
Benzylpenicillin, cephalexin, cefazolin	−	−	−	+	ND
Oleandomycin	−	+	+	+	ND
Streptomycin	+	+	+	−	ND
Tetracycline	−	−	+	+	ND
DNA G + C content (mol%)	39.2	39.3	39.2	39.1	39.1

* All strains were positive for the following tests: respiratory type of metabolism; motility; hydrolysis of aesculin; acid production from sucrose. All strains were negative for the following tests: nitrate reduction; hydrolysis of agar and urea; acid production from arabinose, fructose, mannose, L-rhamnose, ribose, trehalose, N-acetylglucosamine, and glycerol; assimilation of L-arabinose, L-fucose, L-rhamnose, and N-acetylglucosamine. +, positive; −, negative; w, weak reaction; ND, not determined.

**Table 2:** Fatty acid composition (%) of strains belonging to the species *P. distincta* and *P. arctica* A 37-1-2^T^. Strains: 1, KMM 638^T^ [1]; 2, KMM 701 [1]; 3, KMM 3548 [1]; 4, *P. arctica* A 37-1-2^T^ [32].

Fatty Acids *	1	2	3	4
Saturated				
C12:0	tr	2.8	tr	2.4
C14:0	tr	3.9	1.3	1.1
C15:0	4.7	2.5	3.0	2.2
**C16:0**	**15.4**	**18.2**	**16.0**	**12.7**
C17:0	10.3	6.1	5.8	1.5
C18:0	1.8	1.9	1.4	-
Unsaturated				
C15:1 ω8c	4.3	2.7	4.9	1.8
**C16:1 ω7c**	**29.0**	**30.6**	**32.1**	**40.2**
**C17:1 ω8c**	**17.9**	**11.7**	**15.3**	**5.5**
**C18:1 ω7c**	**5.2**	**11.0**	**7.5**	**7.8**
Branched				
iso-C16:0	tr	1.0	tr	-
Hydroxy				
C10:0 3-OH	tr	tr	tr	1.2
C11:0 3-OH	tr	tr	tr	1.1
C12:0 3-OH	6.3	4.8	7.5	6.4
C13:0 3-OH	1.2	tr	1.0	-

* Major components (≥5.0%) are highlighted in bold. tr, trace amount (<1.0%).

## Data Availability

The data presented in this study are openly available in GenBank [National Center for Biotechnology Information (nih.gov)].

## Supplementary Materials

The following supporting information can be downloaded at: https://www.mdpi.com/article/10.3390/ijms24044158/s1.
