# Supplementary material for: Computational Insight into Intraspecies Distinctions in Pseudoalteromonas distincta: Carotenoid-like Synthesis Traits and Genomic Heterogeneity
Source: Int J Mol Sci. 2023 Feb 19;24(4):4158. doi: 10.3390/ijms24044158 (PMC9966250; doi:10.3390/ijms24044158)
Supplement: Supplementary file 1 [file ijms-24-04158-s001.zip › Table S11_ape BGC_identifiers.pdf]

Table S11. Identifiers for genes from the genomic regions containing resorcinol and aryl polyene biosynthetic gene clusters.

Strain	Location	Locus tag	AntiSMASH annotation
P. arctica A 37-1-2T	NZ_CP011026	PARC_RS19935	Transport-related gene
		PARC_RS19940<br>PARC_RS19945<br>PARC_RS19950<br>PARC_RS19955<br>PARC_RS19960	Other genes
		PARC_RS19965	Additional biosynthetic gene
		PARC_RS19970<br>PARC_RS19980	Other genes
		PARC_RS19985	Regulatory gene
		PARC_RS19990	Transport-related gene
		PARC_RS20000	Additional biosynthetic gene
		PARC_RS20005<br>PARC_RS20010<br>PARC_RS20015<br>PARC_RS20020<br>PARC_RS21660<br>PARC_RS20025<br>PARC_RS20030<br>PARC_RS20035<br>PARC_RS20040<br>PARC_RS20045<br>PARC_RS20050	Other genes
		PARC_RS20055	Core biosynthetic gene
		PARC_RS20060<br>PARC_RS20065<br>PARC_RS20070	Other genes
		PARC_RS20075	Additional biosynthetic gene
		PARC_RS20080<br>PARC_RS20085	Transport-related genes
		PARC_RS20090	Other gene
		PARC_RS20095	Core biosynthetic gene
		PARC_RS20100<br>PARC_RS20105	Other genes
		PARC_RS20110<br>PARC_RS20115<br>PARC_RS20120	Additional biosynthetic genes
		PARC_RS20125	Other gene
		PARC_RS20130	Additional biosynthetic gene

		PARC_RS20135	Other gene
		PARC_RS20140	Additional biosynthetic gene
		PARC_RS20145<br>PARC_RS20150<br>PARC_RS20155<br>PARC_RS20160	Other genes
		PARC_RS20165<br>PARC_RS20170	Additional biosynthetic genes
		PARC_RS20175	Other gene
		PARC_RS20180	Core biosynthetic gene
		PARC_RS20185	Other gene
		PARC_RS20190	Additional biosynthetic gene
		PARC_RS20195	Core biosynthetic gene
		PARC_RS20200<br>PARC_RS20205<br>PARC_RS20210	Other genes
		PARC_RS20215	Additional biosynthetic gene
		PARC_RS20220<br>PARC_RS20225<br>PARC_RS20230<br>PARC_RS20235	Other genes
		PARC_RS20240	Additional biosynthetic gene
		PARC_RS20245<br>PARC_RS20250<br>PARC_RS20255	Other genes
		PARC_RS20260	Additional biosynthetic gene
		PARC_RS20265	Regulatory gene
		PARC_RS20270<br>PARC_RS20275<br>PARC_RS20280	Other genes
P. distincta 2-2A-13	NODE_2	NQU47_03185	Transport-related gene
		NQU47_03180<br>NQU47_03175<br>NQU47_03170<br>NQU47_03165<br>NQU47_03160	Other genes
		NQU47_03155	Additional biosynthetic gene

		NQU47_03150	Other gene
		NQU47_03145	Regulatory gene
		NQU47_03140	Transport-related gene
		NQU47_03135	Additional biosynthetic gene
		NQU47_03130<br>NQU47_03125<br>NQU47_03120<br>NQU47_03115<br>NQU47_03110<br>NQU47_03105<br>NQU47_03100<br>NQU47_03095<br>NQU47_03090<br>NQU47_03085<br>NQU47_03080<br>NQU47_03075<br>NQU47_03070	Other genes
		NQU47_03065	Core biosynthetic gene
		NQU47_03060<br>NQU47_03055<br>NQU47_03050<br>NQU47_03045	Other genes
		NQU47_03040	Additional biosynthetic gene
		NQU47_03035<br>NQU47_03030	Transport-related genes
		NQU47_03025	Other gene
		NQU47_03020	Core biosynthetic gene
		NQU47_03015<br>NQU47_03010	Other genes
		NQU47_03005<br>NQU47_03000<br>NQU47_02995	Additional biosynthetic genes
		NQU47_02990	Other gene
		NQU47_02985	Additional biosynthetic gene
		NQU47_02980	Other gene
		NQU47_02975	Additional biosynthetic gene
		NQU47_02970<br>NQU47_02965<br>NQU47_02960	Other genes

		NQU47_02955	
		NQU47_02950<br>NQU47_02945	Additional biosynthetic genes
		NQU47_02940	Other gene
		NQU47_02935	Core biosynthetic gene
		NQU47_02930	Other gene
		NQU47_02925	Additional biosynthetic gene
		NQU47_02920	Core biosynthetic gene
		NQU47_02915<br>NQU47_02910<br>NQU47_02905	Other genes
		NQU47_02900	Additional biosynthetic gene
		NQU47_02895<br>NQU47_02890<br>NQU47_02885	Other genes
		NQU47_02880	Additional biosynthetic gene
		NQU47_02875<br>NQU47_02870<br>NQU47_02865	Other genes
		NQU47_02860	Additional biosynthetic gene
		NQU47_02855	Regulatory gene
		NQU47_02850<br>NQU47_02845<br>NQU47_02840	Other genes
P. paragorgicola KMM 3548^T	NZ_AQHE01000022	PPAR_RS18555	Transport-related gene
		PPAR_RS18560<br>PPAR_RS18565<br>PPAR_RS18570<br>PPAR_RS18575<br>PPAR_RS18580	Other genes
		PPAR_RS18585	Additional biosynthetic gene
		PPAR_RS18590	Other gene
		PPAR_RS18595	Regulatory gene
		PPAR_RS18600	Transport-related gene
		PPAR_RS18605	Additional biosynthetic gene
		PPAR_RS18610	Other genes

		PPAR_RS18615<br>PPAR_RS18620<br>PPAR_RS18625<br>PPAR_RS18630<br>PPAR_RS18635<br>PPAR_RS18640<br>PPAR_RS18645<br>PPAR_RS18650<br>PPAR_RS18655<br>PPAR_RS18660	
		PPAR_RS18665	Core biosynthetic gene
		PPAR_RS18670<br>PPAR_RS18675<br>PPAR_RS18680<br>PPAR_RS18685	Other genes
		PPAR_RS18690	Additional biosynthetic gene
		PPAR_RS18695	Transport-related gene
		PPAR_RS18700<br>PPAR_RS18705	Other genes
		PPAR_RS18710	Core biosynthetic gene
		PPAR_RS18715<br>PPAR_RS18720	Other genes
		PPAR_RS18725<br>PPAR_RS18730<br>PPAR_RS18735	Additional biosynthetic genes
		PPAR_RS18740	Other gene
		PPAR_RS18745	Additional biosynthetic gene
		PPAR_RS18750	Other gene
		PPAR_RS18755	Additional biosynthetic gene
		PPAR_RS18760<br>PPAR_RS18765<br>PPAR_RS18770<br>PPAR_RS18775	Other genes
		PPAR_RS18780<br>PPAR_RS18785	Additional biosynthetic genes
		PPAR_RS18790	Other gene
		PPAR_RS18795	Core biosynthetic gene
		PPAR_RS18800	Other gene
		PPAR_RS18805	Additional biosynthetic gene

		PPAR_RS18810	Core biosynthetic gene
		PPAR_RS18815<br>PPAR_RS18820<br>PPAR_RS18825	Other genes
		PPAR_RS18830	Additional biosynthetic gene
		PPAR_RS18835<br>PPAR_RS18840<br>PPAR_RS18845	Other genes
		PPAR_RS18850	Additional biosynthetic gene
		PPAR_RS18855<br>PPAR_RS18860<br>PPAR_RS18865	Other genes
		PPAR_RS18870	Additional biosynthetic gene
		PPAR_RS18875	Regulatory gene
		PPAR_RS18880<br>PPAR_RS18885<br>PPAR_RS18890	Other genes
P. agarivorans DSM 14585^T	NZ_CP011012	PAGA_RS19690<br>PAGA_RS19695	Other genes
		PAGA_RS19700	Regulatory gene
		PAGA_RS19705	Transport-related gene
		PAGA_RS19710	Additional biosynthetic gene
		PAGA_RS19715<br>PAGA_RS19720<br>PAGA_RS19725<br>PAGA_RS19730	Other genes
		PAGA_RS19735<br>PAGA_RS19740<br>PAGA_RS19745	Additional biosynthetic genes
		PAGA_RS19750	Other gene
		PAGA_RS19755	Additional biosynthetic gene
		PAGA_RS19760	Other gene
		PAGA_RS19765	Additional biosynthetic gene
		PAGA_RS19770<br>PAGA_RS19775<br>PAGA_RS19780<br>PAGA_RS19785	Other genes

		PAGA_RS19790	
		PAGA_RS19795	Additional biosynthetic gene
		PAGA_RS19800	Other gene
		PAGA_RS19805	Core biosynthetic gene
		PAGA_RS19810	Other gene
		PAGA_RS19815	Additional biosynthetic gene
		PAGA_RS19820	Core biosynthetic gene
		PAGA_RS19825<br>PAGA_RS19830<br>PAGA_RS19835<br>PAGA_RS19840<br>PAGA_RS19845<br>PAGA_RS19850	Other genes
		PAGA_RS19855	Additional biosynthetic gene
		PAGA_RS19860<br>PAGA_RS19865<br>PAGA_RS19870	Other genes
		PAGA_RS19875	Additional biosynthetic gene
		PAGA_RS19880	Regulatory gene
		PAGA_RS19885<br>PAGA_RS19890<br>PAGA_RS19895	Other genes
		PAGA_RS19900	Additional biosynthetic gene
"*P. telluritireducens*" DSM 16098	NZ_LVCM01000051	A2I98_RS17895<br>A2I98_RS17900	Other genes
		A2I98_RS17905	Regulatory gene
		A2I98_RS17910	Transport-related gene
		A2I98_RS17915	Additional biosynthetic gene
		A2I98_RS17920<br>A2I98_RS17925<br>A2I98_RS17930<br>A2I98_RS17935	Other genes
		A2I98_RS17940<br>A2I98_RS17945<br>A2I98_RS17950	Additional biosynthetic genes
		A2I98_RS17955	Other gene

		A2I98_RS17960	Additional biosynthetic gene
		A2I98_RS20735<br>A2I98_RS17965	Other genes
		A2I98_RS17970	Additional biosynthetic gene
		A2I98_RS17975<br>A2I98_RS17980<br>A2I98_RS17985<br>A2I98_RS17990<br>A2I98_RS17995	Other genes
		A2I98_RS18000	Additional biosynthetic gene
		A2I98_RS18005	Other gene
		A2I98_RS18010	Core biosynthetic gene
		A2I98_RS18015	Other gene
		A2I98_RS18020	Additional biosynthetic gene
		A2I98_RS18025	Core biosynthetic gene
		A2I98_RS18030<br>A2I98_RS18035<br>A2I98_RS18040<br>A2I98_RS18045<br>A2I98_RS18050	Other genes
		A2I98_RS18055	Additional biosynthetic gene
		A2I98_RS18060<br>A2I98_RS18065<br>A2I98_RS18070	Other genes
		A2I98_RS18075	Additional biosynthetic gene
		A2I98_RS18080	Regulatory gene
		A2I98_RS18085<br>A2I98_RS18090<br>A2I98_RS18095	Other genes
		A2I98_RS18100	Additional biosynthetic gene
		A2I98_RS18105	Other gene
P. nigrifaciens NBRC 103036^T	NZ_BJXZ01000017	PNI02_RS08160	Regulatory gene
		PNI02_RS19015<br>PNI02_RS08165	Other genes
		PNI02_RS08170	Transport-related gene

		PNI02_RS08175<br>PNI02_RS08180<br>PNI02_RS08185	Other genes
		PNI02_RS08190<br>PNI02_RS08195<br>PNI02_RS08200	Additional biosynthetic genes
		PNI02_RS08205	Other gene
		PNI02_RS08210	Additional biosynthetic gene
		PNI02_RS08215	Other gene
		PNI02_RS08220	Additional biosynthetic gene
		PNI02_RS08225<br>PNI02_RS08230<br>PNI02_RS08235<br>PNI02_RS08240<br>PNI02_RS08245	Other genes
		PNI02_RS08250	Additional biosynthetic gene
		PNI02_RS08255	Other gene
		PNI02_RS08260	Core biosynthetic gene
		PNI02_RS08265	Other gene
		PNI02_RS08270	Additional biosynthetic gene
		PNI02_RS08275	Core biosynthetic gene
		PNI02_RS08280<br>PNI02_RS08285<br>PNI02_RS08290<br>PNI02_RS08295<br>PNI02_RS19105<br>PNI02_RS08305<br>PNI02_RS08310<br>PNI02_RS08315	Other genes
		PNI02_RS08320	Additional biosynthetic gene
		PNI02_RS08325	Regulatory gene
		PNI02_RS08330<br>PNI02_RS08335<br>PNI02_RS08340<br>PNI02_RS08345	Other genes
		PNI02_RS08350	Additional biosynthetic gene
		PNI02_RS08355	Other gene

		PNI02_RS08360	Additional biosynthetic gene
P. translucida KMM 520^{T}	NZ_CP011035	PTRA_RS19000<br>PTRA_RS17890	Other genes
		PTRA_RS17895<br>PTRA_RS17900<br>PTRA_RS17905	Regulatory genes
		PTRA_RS17910	Transport-related gene
		PTRA_RS17915<br>PTRA_RS17920<br>PTRA_RS17925	Other genes
		PTRA_RS17930<br>PTRA_RS17935<br>PTRA_RS17940	Additional biosynthetic genes
		PTRA_RS17945	Other gene
		PTRA_RS17950	Additional biosynthetic gene
		PTRA_RS17955	Other gene
		PTRA_RS17960	Additional biosynthetic gene
		PTRA_RS17965<br>PTRA_RS17970<br>PTRA_RS17975<br>PTRA_RS17980<br>PTRA_RS17985	Other genes
		PTRA_RS17990	Additional biosynthetic gene
		PTRA_RS17995	Other gene
		PTRA_RS18000	Core biosynthetic gene
		PTRA_RS18005<br>PTRA_RS18010	Other genes
		PTRA_RS18015	Additional biosynthetic gene
		PTRA_RS18020	Core biosynthetic gene
		PTRA_RS18025<br>PTRA_RS18030<br>PTRA_RS18035<br>PTRA_RS18040	Other genes
		PTRA_RS18045	Additional biosynthetic gene
		PTRA_RS18050<br>PTRA_RS18055<br>PTRA_RS18060	Other genes

		PTRA_RS18065	Additional biosynthetic gene
		PTRA_RS18070	Regulatory gene
		PTRA_RS19310<br>PTRA_RS18075<br>PTRA_RS18080<br>PTRA_RS18085	Other genes
		PTRA_RS18090	Additional biosynthetic gene
P. distincta ANT/505	NZ_ADOP01000007	PH505_RS04320	Additional biosynthetic gene
		PH505_RS04315<br>PH505_RS04310<br>PH505_RS04305<br>PH505_RS04300<br>PH505_RS04295<br>PH505_RS04290<br>PH505_RS04285<br>PH505_RS04280	Other genes
		PH505_RS04275<br>PH505_RS04270<br>PH505_RS04265	Additional biosynthetic genes
		PH505_RS04260	Other gene
		PH505_RS04255	Additional biosynthetic gene
		PH505_RS04250	Other gene
		PH505_RS04245	Additional biosynthetic gene
		PH505_RS04240<br>PH505_RS04235<br>PH505_RS04230<br>PH505_RS04225<br>PH505_RS04220	Other genes
		PH505_RS04215	Additional biosynthetic gene
		PH505_RS04210	Other gene
		PH505_RS04205	Core biosynthetic gene
		PH505_RS04200	Other gene
		PH505_RS04195	Additional biosynthetic gene
		PH505_RS04190	Core biosynthetic gene
		PH505_RS04185	Other gene

		PH505_RS04180	Additional biosynthetic gene
		PH505_RS04175<br>PH505_RS04170<br>PH505_RS04165<br>PH505_RS04160	Other genes
		PH505_RS04155	Additional biosynthetic gene
		PH505_RS04150<br>PH505_RS04145<br>PH505_RS04140	Other genes
		PH505_RS04135	Additional biosynthetic gene
		PH505_RS04130	Regulatory gene
		PH505_RS04125<br>PH505_RS04120<br>PH505_RS04115<br>PH505_RS04110<br>PH505_RS04105	Other genes
Pseudoalteromonas sp. TAE79	NZ_AUTL01000264	L655_RS0120600<br>L655_RS0120605<br>L655_RS0120610<br>L655_RS0120615<br>L655_RS0120620<br>L655_RS0120625	Other genes
		L655_RS0120630<br>L655_RS0120635<br>L655_RS0120640	Additional biosynthetic genes
		L655_RS0120645	Other gene
		L655_RS0120650	Additional biosynthetic gene
		L655_RS0120655	Other gene
		L655_RS0120660	Additional biosynthetic gene
		L655_RS0120665<br>L655_RS0120670<br>L655_RS0120675<br>L655_RS0120680<br>L655_RS0120685	Other genes
		L655_RS0120690	Additional biosynthetic gene
		L655_RS0120695	Other gene
		L655_RS0120700	Core biosynthetic gene

		L655_RS0120705	Other gene
		L655_RS0120710	Additional biosynthetic gene
		L655_RS01000000125975	Core biosynthetic gene
		L655_RS0120720	Other gene
		L655_RS0120725	Additional biosynthetic gene
		L655_RS0120730	Other gene
Pseudoalteromonas sp. TAE80	NZ_AUTM01000285	L657_RS0118550<br>L657_RS1000000127615<br>L657_RS0118545<br>L657_RS0118540<br>L657_RS0118535<br>L657_RS0118530<br>L657_RS0118525	Other genes
		L657_RS0118520<br>L657_RS0118515<br>L657_RS0118510	Additional biosynthetic genes
		L657_RS0118505	Other gene
		L657_RS0118500	Additional biosynthetic gene
		L657_RS0118495	Other gene
		L657_RS0118490	Additional biosynthetic gene
		L657_RS0118485<br>L657_RS0118480<br>L657_RS0118475<br>L657_RS0118470<br>L657_RS0118465	Other genes
		L657_RS0118460	Additional biosynthetic gene
		L657_RS0118455	Other gene
		L657_RS0118450	Core biosynthetic gene
		L657_RS0118445	Other gene
		L657_RS0118440	Additional biosynthetic gene
		L657_RS0118435	Core biosynthetic gene
		L657_RS0118430	Other gene
		L657_RS0118425	Additional biosynthetic gene
		L657_RS0118420	Other gene
