# Supplementary material for: Computational Insight into Intraspecies Distinctions in Pseudoalteromonas distincta: Carotenoid-like Synthesis Traits and Genomic Heterogeneity
Source: Int J Mol Sci. 2023 Feb 19;24(4):4158. doi: 10.3390/ijms24044158 (PMC9966250; doi:10.3390/ijms24044158)
Supplement: Supplementary file 1 [file ijms-24-04158-s001.zip › Table S1_16S rRNA DNA.pdf]

Table S1. The 16S rRNA gene sequences content and similarity for the strains *P. distincta* 16-SW-7, *P. distincta* ATCC 700518^T, *P. distincta* KMM 3548^T, and *P. arctica* A 37-1-2^T (from the alignment on query gene of the strain 16-SW-7 under the locus_tag FFU37_04590)

**Genome Name**	**NCBI Homolog 16S rRNA (Locus_tag)**	**Identity %**	**Genome ID**	**Chromosome (Contig)/ Length**	**Coordinates**
***Pseudoalteromonas distincta* 16-SW-7**	FFU37_04590<br>FFU37_00210<br>FFU37_18380<br>FFU37_15325<br>FFU37_14755<br>FFU37_15610<br>FFU37_14215<br>FFU37_00060	**100.00**<br>**100.00**<br>99.94<br>99.94<br>99.94<br>99.87<br>99.87<br>99.87	CP040558<br>CP040558<br>CP040559<br>CP040558<br>CP040558<br>CP040558<br>CP040558<br>CP040558	L1/3735685<br>L1/ 3735685<br>S1/795760<br>L1/3735685<br>L1/3735685<br>L1/3735685<br>L1/3735685<br>L1/3735685	1023946..1025481<br>48439..49974<br>375599..377134<br>3416198..3417733<br>3277653..3279188<br>3477632..3479167<br>3172769..3174304<br>16934..18469
***Pseudoalteromonas arctica* A 37-1-2^T**	PARC_bR026<br>PARC_aR001<br>PARC_aR004<br>PARC_aR007<br>PARC_aR012<br>PARC_aR016<br>PARC_aR019<br>PARC_aR022<br>PARC_aR025	**100.00**<br>99.61<br>99.67<br>99.28<br>99.34<br>99.67<br>99.28<br>99.67<br>99.67	CP011026<br>CP011025<br>CP011025<br>CP011025<br>CP011025<br>CP011025<br>CP011025<br>CP011025<br>CP011025	Chr II/ 783876<br>Chr I/ 3840834<br>Chr I/ 3840834<br>Chr I/ 3840834<br>Chr I/ 3840834<br>Chr I/ 3840834<br>Chr I/ 3840834<br>Chr I/ 3840834<br>Chr I/ 3840834	192599..194122<br>36983..38506<br>313581..315104<br>345719..347242<br>2483422..2484945<br>3068857..3070380<br>3589860..3591383<br>3690377..3691900<br>3815090..3816613
Pseudoalteromonas distincta KMM 3548 (=P. paragorgicola)	PPAR_aR004<br>PPAR_aR007	99.87<br>**100.00**	AQHE01000014<br>AQHE01000021	14/ 767276<br>21/ 300025	187495..189018<br>298305..299828
Pseudoalteromonas distincta 2-2A-13	NQU47_RS19790	**100.00**	JANIHL010000061	Contig 61/1771	192..1727
Pseudoalteromonas distincta ATCC 700518^T	QT16_19995	99.94	JWIG01000030	C30/ 180150	175393..176935
