# Supplementary material for: Computational Insight into Intraspecies Distinctions in Pseudoalteromonas distincta: Carotenoid-like Synthesis Traits and Genomic Heterogeneity
Source: Int J Mol Sci. 2023 Feb 19;24(4):4158. doi: 10.3390/ijms24044158 (PMC9966250; doi:10.3390/ijms24044158)
Supplement: Supplementary file 1 [file ijms-24-04158-s001.zip › Table S2_accession numbers.pdf]

Table S2. List of accession numbers for genomes used in phylogenetic analyses

Strain	GenBank Accession No.
Alteromonas macleodii ATCC 27126T	GCF_000172635.2
Pseudoalteromonas agarivorans DSM 14585T	*GCF_002310855.1*
Pseudoalteromonas aliena SW19T	*GCF_014905615.1*
Pseudoalteromonas amylolytica JW1T	*GCF_001854605.1*
Pseudoalteromonas arabiensis JCM 17292T	*GCF_001550155.1*
Pseudoalteromonas arctica A 37-1-2T	*GCF_000238395.3*
Pseudoalteromonas aurantia 208T	*GCF_014858725.1*
Pseudoalteromonas byunsanensis JCM 12483T	*GCF_001854475.1*
Pseudoalteromonas caenipelagi JBTF-M23T	*GCF_013140855.1*
Pseudoalteromonas carrageenovora ATCC 43555T	*GCF_900239935.1*
Pseudoalteromonas carrageenovora IAM 12662T	*GCF_014925295.1*
Pseudoalteromonas carrageenovora NBRC 12985T	*GCF_006539245.1*
Pseudoalteromonas citrea DSM 8771T	*GCF_000238375.3*
Pseudoalteromonas denitrificans DSM 6059T	*GCF_900112265.1*
Pseudoalteromonas distincta 16-SW-7	***GCF_005877035.1***
Pseudoalteromonas distincta 2-2A-13	***JANIHL010000000***
Pseudoalteromonas distincta ANT/505	***GCF_000212655.2***
Pseudoalteromonas distincta ATCC 700518T	***GCF_000814675.1***
Pseudoalteromonas distincta U2A	***GCF_008370225.1***
Pseudoalteromonas donghaensis HJ51T	*GCF_003515105.1*
Pseudoalteromonas elyakovii SM1926	***GCF_007786285.1***
Pseudoalteromonas elyakovii VKPM B-3905T	*JANJFO000000000*
Pseudoalteromonas espejiana DSM 9414T	*GCF_002221525.1*
Pseudoalteromonas espejiana NBRC 102222T	*GCF_007989585.1*
Pseudoalteromonas flavipulchra DSM 14401T	*GCF_014858715.1*
Pseudoalteromonas flavipulchra LMG 20361T	*GCF_014596995.1*
Pseudoalteromonas fuliginea KMM 216T	*GCF_000690055.1*
Pseudoalteromonas galatheae S4498T	*GCF_005886105.2*
Pseudoalteromonas gelatinilytica NH153T	*GCF_001641615.1*
Pseudoalteromonas haloplanktis ATCC 14393T	*GCF_000238355.1*
Pseudoalteromonas issachenkonii KCTC 12958	*GCF_001455325.1*
Pseudoalteromonas issachenkonii KMM 3549T	*GCF_002310795.1*
Pseudoalteromonas lipolytica CGMCC 1.8499T	*GCF_900116435.1*
Pseudoalteromonas lipolytica LMEB 39T	*GCF_014925285.1*
Pseudoalteromonas luteoviolacea CH130T	*GCF_014925325.1*
Pseudoalteromonas luteoviolacea DSM 6061T	*GCF_001625655.1*
Pseudoalteromonas maricaloris LMG 19692T	*GCF_012641725.1*
Pseudoalteromonas marina DSM 17587T	*GCF_000238335.3*
Pseudoalteromonas mariniglutinosa NCIMB 1770T	*GCF_021613355.1*
Pseudoalteromonas neustonica PAMC 28425T	*GCF_001653135.1*
Pseudoalteromonas nigrifaciens KMM 661T	*GCF_002221505.1*
Pseudoalteromonas nigrifaciens NBRC 103036T	*GCF_007991255.1*
Pseudoalteromonas nigrifaciens NCTC 10691T	*GCF_900455185.1*
Pseudoalteromonas ostreae hOe-66T	*GCF_018069805.1*
Pseudoalteromonas paragorgicola KMM 3548T	***GCF_014918315.1***
Pseudoalteromonas peptidolytica DSM 14001T	*GCF_012641745.1*
Pseudoalteromonas peptidolytica F12-50-A1T	*GCF_014858745.1*
Pseudoalteromonas peptidolytica NBRC 101021T	*GCF_007989895.1*
Pseudoalteromonas phenolica JCM 21460T	*GCF_014925335.1*

Pseudoalteromonas phenolica KCTC 12086T	*GCF_001444405.1*
Pseudoalteromonas phenolica O-BC30T	*GCF_004103265.1*
Pseudoalteromonas piratica OCN003T	*GCF_000788395.1*
Pseudoalteromonas piscicida ATCC 15057T	*GCF_000382005.1*
Pseudoalteromonas piscicida JCM 20779T	*GCF_000238315.3*
Pseudoalteromonas profundi CGMCC 1.15394T	*GCF_014642695.1*
Pseudoalteromonas prydzensis ACAM 620T	*GCF_014925355.1*
Pseudoalteromonas prydzensis DSM 14232T	*GCF_001661495.1*
Pseudoalteromonas rhizosphaerae RA15T	*GCF_902498845.1*
Pseudoalteromonas rubra DSM 6842T	*GCF_000238295.3*
Pseudoalteromonas ruthenica LMG 19699T	*GCF_008808095.1*
Pseudoalteromonas shioyasakiensis JCM 18891T	*GCF_001550135.1*
***Pseudoalteromonas sp. AC163**	***GCF_000497935.1***
***Pseudoalteromonas sp. TAE79**	***GCF_000498015.1***
***Pseudoalteromonas sp. TAE80**	***GCF_000498035.1***
***Pseudoalteromonas sp. TB25**	***GCF_000497995.1***
"Pseudoalteromonas spiralis" DSM 16099	*GCF_001596815.1*
Pseudoalteromonas spongiae UST010723-006T	*GCF_000238255.3*
"Pseudoalteromonas telluritireducens" DSM 16098	*GCF_001596795.1*
Pseudoalteromonas tetraodonis GFCT	*GCF_002310835.1*
Pseudoalteromonas tetraodonis NBRC 103034T	*GCF_007991235.1*
Pseudoalteromonas translucida KMM 520T	*GCF_001465295.1*
Pseudoalteromonas tunicata D2T	*GCF_000153245.1*
Pseudoalteromonas tunicata D2T	*GCF_003568825.1*
Pseudoalteromonas tunicata DSM 14096T	*GCF_002310815.1*
Pseudoalteromonas ulvae UL12T	*GCF_014925405.1*
Pseudoalteromonas undina DSM 6065T	*GCF_000238275.3*

***The strains belonging to the species *P. distincta* are in bold (this study)**
