# Supplementary material for: Computational Insight into Intraspecies Distinctions in Pseudoalteromonas distincta: Carotenoid-like Synthesis Traits and Genomic Heterogeneity
Source: Int J Mol Sci. 2023 Feb 19;24(4):4158. doi: 10.3390/ijms24044158 (PMC9966250; doi:10.3390/ijms24044158)
Supplement: Supplementary file 1 [file ijms-24-04158-s001.zip › Table S3_P_distincta strains.pdf]

Table S3. The strains belonging to the species *Pseudoalteromonas distincta* according to the genome-based identification by EzBiocloud

GenBank accession number	Original name	Strain	Source	Geographical origin	Country	Year
GCA_000814675.1	*Pseudoalteromonas distincta*	ATCC 700518(Type)	Marine sponge	Bering Sea, 109 miles east of Kamchatka Peninsula	Russia	1989
GCA_000497935.1	*Pseudoalteromonas haloplanktis*	AC163	Antarctic marine sponge	-	-	-
GCA_000497995.1	*Pseudoalteromonas haloplanktis*	TB25	Antarctic marine sponge	-	-	-
GCA_000498015.1	*Pseudoalteromonas haloplanktis*	TAE79	Water column	-	-	-
GCA_000498035.1	*Pseudoalteromonas haloplanktis*	TAE80	Water column	-	-	-
GCA_000212655.3	*Pseudoalteromonas haloplanktis*	ANT/505	-	-	-	-
GCA_005877035.1	*Pseudoalteromonas distincta*	16-SW-7	Seawater	coastline of Kuril Isls, Okhotsk Sea, Pacific Ocean	Russia	2017
GCA_007786285.1	*Pseudoalteromonas elyakovii*	SM1926	-	-	-	2017
GCA_008370225.1	*Pseudoalteromonas distincta*	U2A	Algae surface(host:Fucus)	Willows Beach, Victoria, BC	Canada	2012
GCA_000803085.1	*Pseudoalteromonas flavipulchra NCIMB 2033 = ATCC BAA-314*	ATCC BAA-314	Sea Water	Nice	France	1978
