# Supplementary material for: Computational Insight into Intraspecies Distinctions in Pseudoalteromonas distincta: Carotenoid-like Synthesis Traits and Genomic Heterogeneity
Source: Int J Mol Sci. 2023 Feb 19;24(4):4158. doi: 10.3390/ijms24044158 (PMC9966250; doi:10.3390/ijms24044158)
Supplement: Supplementary file 1 [file ijms-24-04158-s001.zip › Table S9_Streptorubin_MEP.pdf]

Table S9. Prediction of MetaCyc pathways for streptorubin B biosynthesis (PWY1A0-6120), methylerythritol phosphate pathway II (PWY-7560), and 4,4'-diapolycopenedioate biosynthesis (PWY-6767) in *P. distincta** and *P. arctica* A 37-1-2^T

**PWY1A0-6120**	**2.3.1.180**	**4.2.1.59/4.2.1.58/4.2.1.60/4.2.1.61****	**1.1.1.100**	**2.3.1.179/2.3.1.41****	**1.3.8.14**	**4.2.1.59/4.2.1.58/4.2.1.60/4.2.1.61****	**2.3.1.179/2.3.1.41****	**1.3.1.9**
Direction	<	>	>	>	<	<	<	<
Enzyme	beta-ketoacyl-[acyl-carrier-protein] synthase III	(3R)-3-hydroxybutanoyl-[acyl-carrier-protein] hydratase	3-hydroxyhexanoyl-[acyl-carrier protein] reductase	3-oxo-decanoyl-[acyl-carrier protein] synthase	L-prolyl-[peptidyl-carrier protein] dehydrogenase	(3R)-3-hydroxybutanoyl-[acyl-carrier-protein] hydratase	3-oxo-decanoyl-[acyl-carrier protein] synthase	trans dodec-2-enoyl-[acp] reductase
Coordinates*	614287..613340	891550..891996	1196519..1197253	1197966..1199195	1463979..1462870	1498431..1497928	2326647..2325451	25176842516524
**PWY-7560 PWY-6767*****	**2.2.1.7/4.1.3.37**	**2.7.7.60**	**4.6.1.12**	**1.17.7.1/1.17.4.3**	**2.7.1.148**	**1.17.7.4/1.17.1.2**	**1.1.1.267**	**1.2.99.10****
Direction	>	>	>	>	>	>	<	>
Enzyme	1-deoxy-D-xylulose 5-phosphate synthase	4-diphosphocytidyl-2C-methyl-D-erythritol synthase	2C-methyl-D-erythritol 2,4-cyclodiphosphate synthaseu	4-hydroxy-3-methylbut-2-en-1-yl diphosphate synthase	4-diphosphocytidyl-2-C-methyl-D-erythritol kinase	dimethylallyl-diphosphate: NAD(P)+ oxidoreductase	1-deoxy-D-xylulose 5-phosphate reductoisomerase	4,4'-diapolycopenedial synthase
Coordinates*	8409..10286	24085..24753	24765..25247	33428..34465	42401..43246	54265..55209	105366..104299	NODE_4_length_233017_cov_73.836012

* - related to the *P. distincta* 2-2A-13 genome (GenBank: JANIHL010000000); ** - duplicated key genes; *** - related to MetaCyc Reaction ID: RXN-12225 (PWY-6767).
